# Lactylation at H3K18 drives pathological angiogenesis via metabolic-epigenetic crosstalk in ischemic retinopathy

**DOI:** 10.7150/thno.136054

**Published:** 2026-08-12

**Authors:** Xin Chen, Yuhan Jiang, Weiqi Qian, Yi Lei, Siyue Chen, Yutian Zhang, Minghui Liang, Yuming Liu, Xiaohong Wang, Ding Ai, Xuyang Yao, Hua Yan

**Affiliations:** 1 School of Medicine, Nankai University, Tianjin, 300071, China.; 2 Department of Ophthalmology, Tianjin Medical University General Hospital, Ministry of Education International Joint Laboratory of Ocular Diseases, Tianjin Key Laboratory of Ocular Trauma, Tianjin Institute of Eye Health and Eye Diseases, Laboratory of Molecular Ophthalmology, Tianjin Medical University, Tianjin, 300070, China.; 3 Tianjin Key Laboratory of Ion and Molecular Function of Cardiovascular Diseases, Tianjin Institute of Cardiology, State Key Laboratory of Experimental Hematology, National Clinical Research Center for Blood Diseases, Key Laboratory of Immune Microenvironment and Disease (Ministry of Education), Tianjin Medical University, Tianjin, 300070, China.; 4 Department of Physiology and Pathophysiology, Tianjin Medical University, 22 Qixiangtai Road, Tianjin 300070, China.; 5 Department of Pharmacology, Tianjin Key Laboratory of Inflammation Biology, State Key Laboratory of Experimental Hematology, School of Basic Medical Sciences, Tianjin Medical University, Tianjin, China.; 6 Department of Genetics, School of Basic Medical Sciences, Tianjin Medical University, Tianjin, 300070, China.

**Keywords:** histone lactylation, angiogenesis, hypoxia, retinal microvascular endothelial cells, oxygen-induced retinopathy

## Abstract

Ocular neovascular disease, characterized by aberrant angiogenesis in the eye, is a primary cause of global vision impairment and blindness. As the primary barrier exposed to hypoxia-related blood metabolites, endothelial cells (ECs) undergo metabolic reprogramming that drives pathological angiogenesis. However, the epigenetic mechanisms that link EC metabolic dysfunction to retinal vasculopathy remain elusive.

**Methods:**

Using western blotting and immunofluorescence analysis of retinal sections/whole-mounts, we confirmed increased histone 3 lactylation at lysine 18 (H3K18la). We subsequently identified downstream target genes through integrated CUT&Tag and RNA sequencing (RNA-seq), assessed their angiogenic regulatory functions using siRNA, and validated the mechanisms *in vivo* employing adeno-associated virus (AAV)-based gene transfer.

**Results:**

Our data indicated that histone lactylation levels were elevated in retinal vascular ECs under hypoxic conditions both *in vivo* and *in vitro*. In oxygen-induced retinopathy (OIR) retinal vascular ECs, H3K18la was the most prominent modification. Pharmacological inhibition of glycolysis suppressed H3K18la levels, concurrently abrogating EC activation and neovascularization. Combined CUT&Tag and RNA-seq analyses revealed that ETS1 was a direct transcriptional target governed by H3K18la in retinal ECs. Silencing ETS1 substantially inhibited hypoxia-induced proliferation, migration, sprouting, and tube formation in human retinal microvascular endothelial cells (HRMECs). Crucially, *in vivo* rescue experiments confirmed that ETS1 overexpression reversed the suppression of pathological neovascularization in OIR mice treated with AAV-Pfkfb3-RNAi.

**Conclusions:**

Collectively, this study revealed a lactate-driven epigenetic cascade wherein H3K18la licenses ETS1-dependent pathological angiogenesis, providing a promising therapeutic avenue for ischemic retinal diseases.

## Introduction

Ocular neovascular diseases, characterized by the pathological formation of immature blood vessels, are the leading causes of vision impairment and blindness worldwide. These conditions include proliferative diabetic retinopathy (PDR), age-related macular degeneration (AMD), retinopathy of prematurity (ROP), and retinal vein occlusion (RVO). Current clinical management relies primarily on intravitreal injections of anti-vascular endothelial growth factor (VEGF) agents to inhibit vascular leakage and neovascularization. However, significant limitations are associated with this therapeutic approach 1-3. Consequently, attaining a thorough understanding of the intrinsic regulatory mechanisms is critical for developing novel diagnostic and therapeutic strategies with significant clinical value.

Higher organisms rely on closed cardiovascular systems in which blood vessels deliver oxygen and nutrients to sustain tissue function. Consequently, vascular diseases pose a major threat to human health 4, 5. Angiogenesis is a complex process in which vascular endothelial cells (ECs) proliferate, migrate, and form new vessels from preexisting vasculature. Dysregulation of this process has been implicated in several pathological conditions. The VEGF signaling pathway has been established as a central regulator orchestrating angiogenic processes 6-8. Emerging evidence indicates that the metabolic reprogramming of ECs also critically drives angiogenesis 9, 10. ECs are directly exposed to the circulating blood as the innermost lining of blood vessels, enabling nutrient sensing and adaptive responses. ECs exhibit distinct metabolic profiles, particularly high glycolytic flux. Their glucose consumption rates rival those of cancer cells, with glycolysis generating approximately 85% of the total ATP 11. Key glycolytic regulators, including *PFKFB3*, *ADORA2A*, and *HK2*, modulate EC energy homeostasis and angiogenesis 11-13. However, the mechanisms linking aberrant metabolic signaling to protein modification and transcriptional reprogramming in metabolically driven vasculopathies remain unclear. Understanding these mechanisms is crucial for disease pathogenesis and novel therapies.

No longer considered as a byproduct of anaerobic metabolism, lactate is increasingly being explored as a signaling molecule. The accumulation of lactate in the tissue microenvironment is a hallmark feature of inflammatory diseases and tumors. Lactate enters cells through monocarboxylate transporters (MCTs) and is sensed extracellularly by lactate receptor GPR81 (HCAR1), subsequently modulating energy metabolism 14, lipolysis 15, and inflammatory responses 16.

Identified as a novel post-translational modification (PTM), protein lactylation involves the covalent addition of lactyl groups to lysine residues 17. Using lipopolysaccharide (LPS)-challenged macrophages to model bacterial infection, the researchers observed enhanced glycolysis with consequent lactate accumulation. This led to the identification of lactyl group conjugation to ε-amino groups of lysine residues within histone N-terminal tails, termed histone lysine lactylation (Kla) 17. This modification mediates key biological processes, including metabolic reprogramming, wound healing, embryonic development, cellular differentiation, and immunosuppression 18.

Aberrant histone PTMs, including methylation and acetylation, constitute established pathogenic mechanisms underlying diverse diseases, such as cancer 19, metabolic disorders 20, and neurodegenerative conditions 21. Functionally analogous to histone acetylation, Kla epigenetically regulates gene transcription 17, 22. Emerging evidence has demonstrated a regulatory role of lactylation in vascular homeostasis 23. Atherosclerosis, a chronic vasculopathy characterized by metabolic dysfunction, progresses via the senescence of vascular smooth muscle cells (VSMC). In atherosclerotic lesions, lactate accumulation drives histone H4 lysine 12 lactylation (H4K12la), which epigenetically activates the transcription of senescence-associated secretory phenotype (SASP) genes, thereby accelerating disease progression 24. Medial arterial calcification (MAC), a distinct vasculopathy involving progressive mineralization of the tunica media, is characterized by lactate-induced H3K18la in VSMCs. This modification upregulates PHOSPHO1 expression and potentiates arterial calcification 25. Pulmonary arterial hypertension (PAH), characterized by right heart failure and pathological proliferation of pulmonary arterial smooth muscle cells (PASMCs), involves lactate-mediated remodeling 26. Mechanistically, the inhibition of pyruvate dehydrogenase kinase (PDK) suppresses histone lactylation, thereby attenuating PAH pathogenesis 27. The potential involvement of endothelial lactylation in ischemic retinopathy, particularly its effect on endothelial dysfunction and pathological angiogenesis under hypoxic conditions, remains unexplored.

Herein, we integrated *in vivo* OIR modeling, *in vitro* hypoxic assays, and CUT&Tag profiling to delineate the mechanistic contribution of lactylation to pathological angiogenesis. We further demonstrated that the targeted attenuation of glycolytic flux reduced pathological neovascularization in retinal vasculopathy by suppressing lactylation-mediated pathways.

## Methods

### Experiments in human retinal microvascular endothelial cells (HRMECs)

HRMECs were obtained from Procell Co., Ltd. HRMECs from passages four to six were used in this study. HRMECs were cultured in endothelial cell medium (ECM) (SC-1001, ScienCell, USA) at 37 °C / 5% CO₂ under humidified conditions. The ECM was prepared by supplementing the basal medium with 5% FBS (0025, ScienCell), 1% ECGS (1052, ScienCell), and 1% penicillin/streptomycin (P/S) (0503, ScienCell). Treatments included 20 mM sodium dichloroacetate (DCA) (RO52025, RHAWN, China), 20 mM sodium oxamate (S26990, Acmec-e, China), 10 mM sodium lactate (S108838, Aladdin, China), 10 µM 3PO (SML1343, Sigma-Aldrich, USA), and 10 µM C646 (HY-13823, MCE, USA). For *in vitro* hypoxia exposure, HRMECs seeded in culture dishes were incubated under 1% O₂ using a Heracell™ 150i CO₂ incubator (Thermo Fisher Scientific, USA).

### siRNA transfection

HRMECs were plated in six-well dishes. At approximately 70–80% confluence, transfection with siRNA was conducted with Lipofectamine™ RNAiMAX (13778150, Thermo Fisher Scientific). Transfections were performed using 50 nM siRNA (sequences provided in Table S1), and functional assays were conducted 48 h post-transfection. *ETS1-*, *PFKFB3*-, and *P300-*targeting siRNAs with scrambled control siRNA were designed by Tsingke Biotechnology (Beijing, China).

### Protein extraction and western immunoblotting

Tissue and cell lysates were extracted in RIPA lysis. Protein levels were assessed with a BCA assay kit (P0010, Beyotime Biotechnology, China), and sample volumes for subsequent western blot analysis were calculated based on measured concentrations. Proteins were electrophoresed by SDS-PAGE, transferred to nitrocellulose (NC) membranes, and blocked with 5% non-fat milk for 1 h. Membranes were then incubated overnight at 4 ºC with the primary antibodies at recommended concentrations (dilutions in Table S2). Membranes were subjected to three TBST washes of 10 min each on the next day, followed by secondary antibody incubation for 1 h and three more TBST washes of 10 min. Finally, the NC membrane was imaged using a Tanon chemiluminescence system (China), followed by signal quantification using the ImageJ software (version 2.16, USA).

### RNA extraction and real-time quantitative PCR analysis

Total RNA was isolated from retinal tissues using TRIzol™ Reagent (15596026CN, Thermo Fisher Scientific). cDNA was generated from total RNA with the EasyScript All-in-One First-Strand cDNA Synthesis SuperMix (AE341-02, TransGen Biotech, China). Gene expression analysis was conducted using SYBR Green reagent (Vazyme, China) on an ABI 7900HT Real-Time PCR System (Life Technologies, USA). Expression levels were normalized to reference genes and quantified by the 2^-ΔΔC^_t_ method. Beta-actin was the internal control for normalization. All primers are listed in Table S3.

### Animal experiments

All animal work was performed in accordance with NIH guidelines for the care and use of laboratory animals (8th edition). Prior to conducting the study, the research protocol received approval from Tianjin Medical University’s Institutional Animal Care and Use Committee. C57BL/6J pups of both sexes were randomized into: (1) vehicle control, (2) DCA, and (3) sodium lactate groups (n = 4–6 per group) 28, 29. Freshly prepared DCA (200 mg/kg/day) and sodium lactate (200 mg/kg/day) were dissolved in PBS. Daily intraperitoneal (i.p.) injections were administered from postnatal days 13 to 16 (P13–P16). Vehicle control pups received PBS at equivalent injection volumes. At postnatal day 17 (P17), mice were anesthetized with avertin (2,2,2-tribromoethanol dissolved in water, Sigma-Aldrich, 0.36 mg/g body weight, i.p.) with pedal withdrawal reflex assessed every 5 min. The retinas were dissected for analysis. C57BL/6J mice were housed in specific pathogen-free (SPF) facilities. Environmental parameters were controlled at 22 ± 1 °C temperature and 50 ± 5% relative humidity, with 12-h light and dark periods. Animal health was monitored quarterly using sentinel animals and all animals remained clinically normal throughout the study period. The animals were handled gently and allowed to explore the experimental environment before the procedures.

### OIR in mice

The OIR mouse model is an established experimental system that mimics ROP and recapitulates key pathological features, including pathological neovascularization and retinal ischemia-reperfusion injury 30. In the OIR model, sex exhibited no discernible effect on the experimental outcomes; consequently, male and female animals were included 30, 31. C57BL/6J mouse pups were designated as postnatal day 0 (P0) at birth. Nursing mothers and pups received *ad libitum* access to food and water from P0 to P7. From P7 to P12, the nursing mothers and pups underwent continuous 75 O₂ exposure. At P13, the mice were returned to normoxic conditions and maintained until P17. Body weight was used to identify and exclude underdeveloped pups, thereby eliminating weight-gain variability as a confounding factor 32. The pups were sacrificed at P17, and the retinas were dissected for downstream analysis 33.

### Immunofluorescence staining

HRMECs were fixed with 4% paraformaldehyde (PFA) (15 min), washed with PBS (3 × 5 min), and permeabilized with 0.5% Triton X-100/PBS (10 min). Blocking was performed with 5% goat serum (30 min). Primary antibodies were applied overnight at 4 °C. After washing with PBS, the cells were incubated with secondary antibodies for 1 h (dark). Nuclear counterstaining was performed with DAPI. Slides were imaged using a confocal microscope (LSM 900; Carl Zeiss, Germany).

The eyes were fixed in 4% PFA at 4 °C for 2 h, then dehydrated in 30% sucrose (4 °C, overnight). Tissues were embedded in optimal cutting temperature compound (OCT) and sectioned at 12 μm using a freezing microtome (Leica CM1950, Germany). Sections mounted on adhesive slides were washed 3 × 5 min with PBS on an orbital shaker to remove residual OCT. After permeabilization with 0.3% Triton X-100 in PBS (RT, 15 min), blocking was performed using 5% goat serum (RT, 1 h). Primary antibodies were applied overnight at 4 °C. Following 3 × 5 min PBS washes (RT), the sections were incubated with species-matched Alexa Fluor-conjugated secondary antibodies (1:400, RT, 2 h). Nuclei were counterstained with DAPI before mounting with antifade medium.

Retinas dissected from PFA-fixed eyes (4 °C, 2 h) underwent permeabilization (1% Triton X-100/PBS, 4 °C, overnight), blocking (5% goat serum, RT, 12 h), and incubation with primary antibodies (1:100, 20 μL/retina, 48 h, 4 °C). After thorough PBS washing, the retinal tissues were placed in secondary antibody solutions (1:300) with optional isolectin B4 (IsoB4) and DAPI. Tissues were washed (PBS, 3 × 30 min, RT; overnight 4 °C in the dark), quartered, and mounted with vitreous side up. Confocal laser scanning microscopy was performed with the LSM 800 system (LSM 800; Carl Zeiss).

Antibodies utilized in immunofluorescence staining procedures are summarized in Table S2.

### Measurement of lactate levels

Intracellular and tissue L-lactate concentrations were quantified using an L-Lactic Acid Assay Kit (BC2235, Solarbio, China), following NADH-coupled enzymatic reactions. Tissues and cells were added to extraction buffer I according to the appropriate ratio, homogenized, sonicated in an ice bath, and centrifuged. The supernatant was added to extraction buffer II, centrifuged, and collected for measurement. The microplate reader was preheated, the wavelength was adjusted to 570 nm, the standard solution was diluted, and the measurements were performed. After measurements, lactic acid content was calculated in accordance with the kit protocol.

### CUT&Tag

Cell/nucleus samples were bound to concanavalin A-conjugated beads for 10 min. Following bead collection, samples were treated with anti-H3K18la antibody (1:50, PTM-1427RM, PTM Biolabs, China) overnight at 4 °C. Secondary antibodies and protein A/G-Tn5 transposase were subsequently added, enabling the targeted fragmentation of protein-associated DNA. During the Tn5-mediated fragmentation, adapter sequences were simultaneously added to both ends of the cleaved fragment. Amplified libraries were subjected to paired-end sequencing using Illumina NovaSeq. Standard protocols from the manufacturer (TD903, Vazyme) were followed with modifications as specified. Samples were quality-controlled using FastQC (version 0.12.1, UK) to remove low-quality reads. The paired-end reads were aligned to the human reference genome (GRCh38/hg38) using Bowtie2 (version 2.4.5). Peaks were detected using ‘-q 5e-2 -f BAMPE’ by MACS2 (version 2.2.9.1, USA). deepTools (version 3.5.4) bamCoverage was used to generate BigWig files with reads per genome coverage (RPGC) normalization, which were visualized using the Integrative Genomics Viewer (IGV, version 0.12.9) software.

### CUT&RUN–qPCR

All experiments were conducted utilizing a Hyperactive pG-MNase CUT&RUN Assay Kit (HD101; Vazyme). Cells were added to the ConA bead suspension and mixed gently. The mixture was incubated for 10 minutes. Primary antibodies (anti-H3K18la, PTM-1427RM, PTM Biolabs; anti-H3K27ac, ab4729, Abcam) were added and incubated overnight at 4°C. The pG-MNase enzyme bound to the primary antibodies was then added, enabling targeted cleavage of DNA sequences in the vicinity of the target protein. Released DNA fragments were purified. The purified products were used for subsequent qPCR to detect target protein–DNA interactions. Primer sequences used in this study are listed in Table S4.

### Scratch wound healing assay

Cells were seeded in six-well plates and cultured to 80–90% confluency in complete growth medium. The procedure was carried out with one disinfection-treated 200 μL tip. Straight scratches were generated perpendicular to the bottom of the plate. After three washes with sterile PBS to remove debris, antiproliferative treatment with mitomycin C (5 μg/mL, HY-13316, MCE) was performed using serum-depleted culture medium. The plates were cultured for 24 h under standard humidified conditions. Scratch widths were quantified using ImageJ software.

### Tube formation assay

Corning® Matrigel® (354234, USA) was thawed overnight at 4 °C. An equal volume of ice-cold serum-free ECM was added to achieve a 1:1 dilution. The diluted matrix solution (150 μL/well) was coated onto 24-well plates followed by gelation at 37 °C for 40 min. HRMECs (5 × 10⁴ cells/well) were seeded onto polymerized matrices. The cells were imaged using a Zeiss Axiovert microscope after 6 h. Tube formation was quantified using ImageJ software (Angiogenesis Analyzer plugin).

### Spheroid sprouting assay

HRMEC-coated Cytodex-3 beads (GE Healthcare, USA) were incubated with HRMECs (200 cells/bead) for 4 h to establish cell-bead adhesion. Fibrinogen solution (F8630, Sigma-Aldrich) was reconstituted at 2.5 mg/mL in ECM, then mixed with 0.5 U/mL thrombin (T4648, Sigma-Aldrich) and 50 μg/mL aprotinin (A1153, Sigma-Aldrich). Cell-bound beads were embedded in the fibrin matrix (500 μL/well) within 48-well plates. After matrix polymerization (20 min), angiogenic stimuli were applied. After 72 h of culture, the specimens were fixed with 4% PFA, permeabilized with 0.5% Triton X-100, blocked with 5% goat serum, and stained with phalloidin (P1951, Sigma-Aldrich) (1:200 dilution) for 2 h. The confocal laser scanning microscope (LSM 800, Carl Zeiss) was used to acquire z-stack images. Vessel sprouting was analyzed employing the ImageJ platform.

### Immunoprecipitation (IP)

HRMECs from a 10-cm culture dish (~3 × 10^6^ cells per IP sample) were harvested. Lysates were immunoprecipitated using target-specific or isotype-control IgG antibodies at 4 °C for 12 h with gentle agitation. Protein A/G agarose beads (sc-2003, Santa Cruz, USA; 20 μL bead slurry) were then added and incubated at RT with rotation for 2 h. Bead complexes were washed five times with an IP lysis buffer. Following the final wash, the bead-bound proteins were centrifuged (2000 × g), resuspended in the SDS loading buffer, and denatured at 95 °C for 5 min prior to SDS-PAGE and immunoblotting analysis.

### Enrichment analysis

Statistically significant binding peaks were defined as FDR < 0.05. Functional enrichment analyses of the Gene Ontology Biological Process (GO-BP) and KEGG pathways were conducted using clusterProfiler (version 4.8.2, China). Significant pathways were identified with Benjamini-Hochberg adjusted *P* values (adj. *P*) less than 0.05.

Hypoxia-related genes were extracted from the hallmark gene set in the Molecular Signatures Database version 7.0 (MSigDB; www.gsea-msigdb.org, USA), which includes 200 hypoxia genes. Transcriptional profiles of murine OIR retinal tissue at P17 are available on the GEO Accession viewer (nih.gov) (GSE130400). Time-series paired ATAC-seq data in human umbilical ECs from normoxic to hypoxic conditions are available on the GEO Accession viewer (nih.gov) (GSE145774).

### Statistical analysis

Animals were randomly allocated to groups before blinded data analysis. Housing conditions, diet, and handling procedures were identical across the groups. All the data were obtained from biological replicates. Following normality confirmation via Shapiro–Wilk testing, parametric comparisons were performed with two-tailed unpaired *t*-tests (two groups) or one-way ANOVA and two-way ANOVA with Tukey’s multiple comparison test (≥ 3 groups). The Mann–Whitney U test or Kruskal–Wallis test, followed by Dunn’s multiple comparison test, was used for non-normally distributed data, where appropriate. Significance was defined as *P* < 0.05, using the following asterisks: *P < 0.05, **P < 0.01, ***P < 0.001, ****P < 0.0001; NS, not significant (*P* < 0.05). Figure legends for each experiment indicate the statistical tests used. Statistical analyses were performed using GraphPad Prism 8.0 (GraphPad Software, San Diego, CA, USA).

## Results

### H3K18 lactylation-mediated metabolic reprogramming is associated with retinal angiogenesis in OIR

Physiological and pathological neovascularization are both dependent on the angiogenic process. Hypoxia-driven pathological angiogenesis is a hallmark of ischemic retinopathies, such as diabetic retinopathy (DR) and ROP, and is characterized by retinal vascular EC dysfunction, vascular leakage (manifesting as exudates and hemorrhages), and compensatory neovascularization 34-36. This process disrupts oxygen/nutrient delivery, creates metabolic supply-demand imbalances, and impairs neuroretinal function 37. The OIR mouse model is widely used to study retinopathy of prematurity and PDR 30, 38. Recent studies indicated that pathological neovascularization involves endothelial metabolic reprogramming, notably enhancing glycolysis, which is the primary lactate-producing pathway 39, 40.

**Figure 1:**
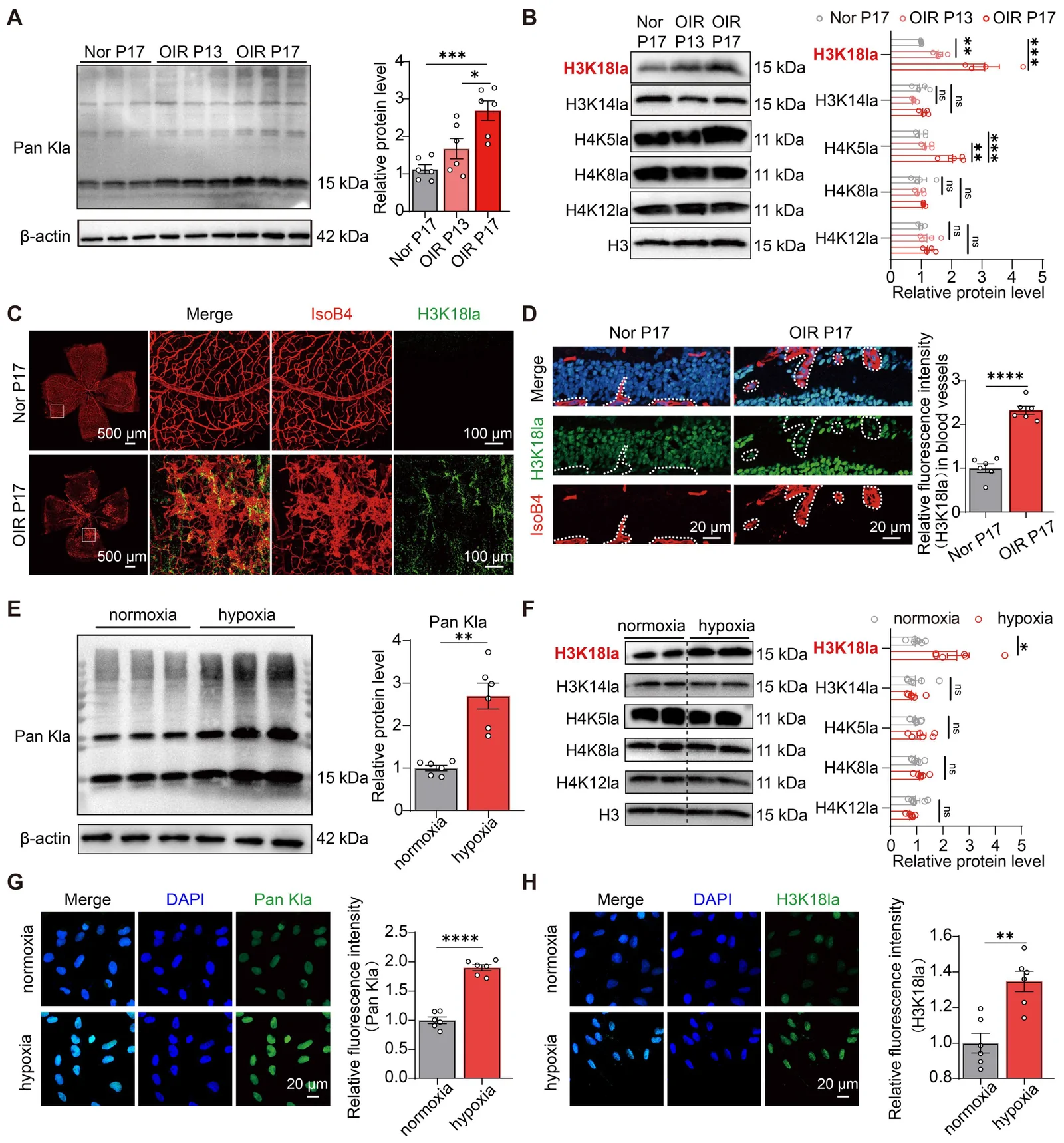
** Elevated lactylation levels are associated with retinal neovascularization in oxygen-induced retinopathy (OIR) (A)** Western blot analysis of pan-lysine lactylation (pan-Kla) in the OIR mouse model (n = 6). **(B)** Immunoblots of site-specific histone lactylation marks (H3K14la, H3K18la, H4K5la,H4K8la, and H4K12la) in the retinas of normoxic controls (Nor P17) and OIR mice at P13 and P17 (n = 4). **(C)** Representative retinal whole-mounts costained for H3K18la (green) and IsoB4 (red) showing H3K18la expression in normoxic controls (Nor P17) and OIR mice at P17. Scale bars: 500 µm (left panels); 100 µm (right panels).** (D)** Immunofluorescence staining of retinal sections revealed upregulated H3K18la in the retinal vasculature of OIR P17 mice compared with that in normoxic controls (Nor P17). Scale bar: 20 µm (n = 6). **(E)** Immunoblots of pan-lysine lactylation in human retinal microvascular endothelial cells (HRMECs) under hypoxic (1% O_2_) and normoxic conditions (n = 6). **(F)** Site-specific histone lactylation marks (H3K14la, H3K18la, H4K5la, H4K8la, and H4K12la) in HRMECs treated for 24 h with hypoxic (1% O_2_) or normoxic (n = 6) conditions. **(G,H)** Immunofluorescence analysis of pan-lysine lactylation (green) and H3K18la (green) in HRMECs under hypoxic conditions. Scale bar: 20 µm (n = 6). Statistical analyses were performed using one-way ANOVA with Tukey’s multiple comparison test (A, B:H3K14la, H3K18la, H4K5la, and H4K12la), Kruskal–Wallis test followed by Dunn’s multiple comparison test (B:H4K8la), or unpaired two-tailed *t*-test (D-H). Data represent means ± SEM; *P < 0.05, **P < 0.01, ***P < 0.001, ****P < 0.0001.

To determine whether lactate levels increased in retinal vascular ECs during pathological angiogenesis, we measured retinal lactate levels in normoxic and OIR mice on P17 using a lactate assay kit. Retinal lactate concentrations were elevated in OIR mice relative to normoxic controls (Figure S1A). As lactate is a precursor for protein lactylation 17, we hypothesized that lactylation would be elevated in OIR. Western blotting revealed higher global lactylation in OIR retinas than in normoxic controls, which was predominantly localized to histones (Figure 1A). Among histone lactylations, H3K18la exhibited the most significant increase (Figure 1B). Immunofluorescence staining of both retinal flat mounts and sections confirmed elevated global lactylation (Figure S1B-C) and H3K18la (Figure 1C-D) in the retinal vascular system of OIR mice versus controls. Hypoxia plays a vital role in retinal vasculopathies 41. *In vitro*, we exposed HRMECs to 1% O_2_ for 24 h to mimic the ischemic retinopathy microenvironment. Western blot analysis of cell lysates showed increased HIF-1α levels (Figure S1D). Significant increases in global lactylation (Figure 1E) and H3K18la levels (Figure 1F) were observed. These findings were corroborated by immunofluorescence analysis (Figure 1G-H).

Collectively, these data suggest concurrent upregulation of lactate and histone hyperlactylation, most prominently at H3K18, under both *in vivo* ischemic retinopathy and *in vitro* hypoxic conditions, indicating lactate accumulation as a biochemical precursor of aberrant histone modification during pathological angiogenesis.

### H3K18 hyperlactylation escalates pathologic neovessel formation

**Figure 2:**
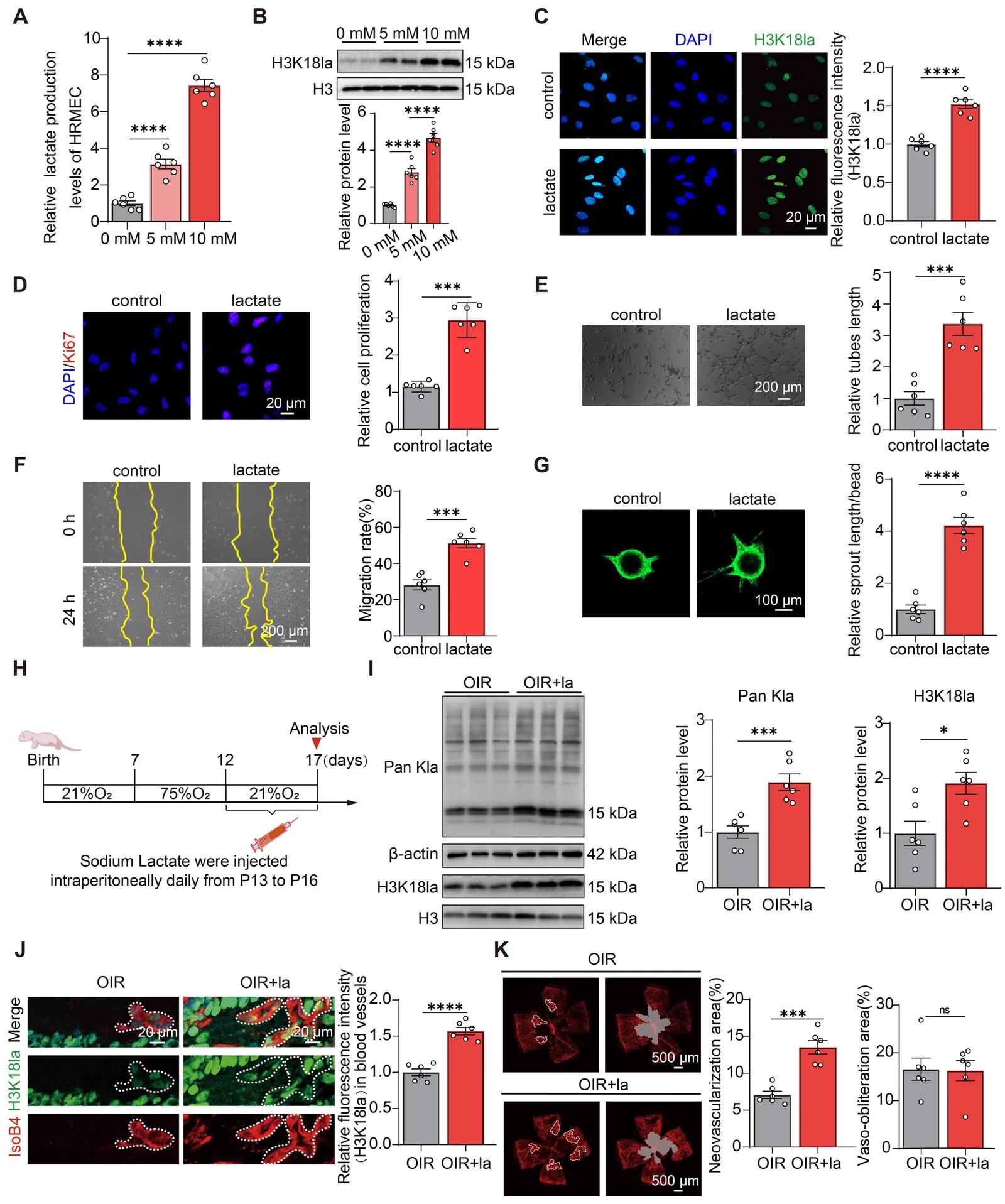
** Histone hyperlactylation promotes angiogenesis *in vivo* and *in vitro.* (A)** Intracellular lactate levels in HRMECs after lactate supplementation, as measured by a colorimetric assay (n = 6). **(B)** Representative immunoblots and quantification of H3K18la protein levels in HRMECs treated with graded lactate concentrations (0, 5, and 10 mM) (n = 6). **(C)** Representative immunofluorescence images of H3K18la (green) among lactate-treated HRMECs. Scale bar: 20 µm (n = 6). **(D)** Representative immunofluorescence images of Ki67 (red) and DAPI (blue) in HRMECs. Scale bar: 20 µm (n = 6). **(E)** Tube formation assay of HRMECs. Scale bar: 200 µm (n = 6). **(F)** Scratch wound healing assay in HRMECs. Scale bar: 200 µm (n = 6). **(G)** Spheroid sprouting assay using HRMECs. Scale bar: 100 µm (n = 6). **(H)** Schematic of the OIR model establishment and lactate administration protocol (200 mg/kg) via daily intraperitoneal injection (P13–P16). **(I)** Western blot analysis of pan-Kla and H3K18la levels in the retinas (n = 6 per group). **(J)** Immunofluorescence analysis of retinal cryosections showing elevated H3K18la levels in the vasculature of lactate-treated OIR mice compared with those in controls. Scale bar: 20 µm (n = 6). **(K)** Representative retinal whole-mounts after saline or lactate i.p. injection with quantification of neovascular and avascular zones (scale bar: 500 µm). (Neovascularization analysis, n = 6; avascular quantification, n = 6). Statistical significance was determined using a one-way ANOVA with Tukey’s multiple comparison test (A,B) or a two-tailed unpaired Student’s *t*-test (C-G,I-K). Data presented as mean ± SEM (*P < 0.05, **P < 0.01, ***P < 0.001, ****P < 0.0001).

To investigate the functional impact of lactylation on pathological angiogenesis, HRMECs were treated with sodium lactate to elevate global lactylation levels. Lactate treatment dose-dependently increased intracellular lactate (Figure 2A) and histone lactylation levels in HRMECs, as validated by western blotting and immunofluorescence staining (Figure 2B-C, S2A-B). Functional assays revealed that lactate significantly enhanced the angiogenic capacity, as evidenced by increased proliferation (Figure 2D), improved tube formation (Figure 2E), enhanced migration (Figure 2F), and spheroid sprouting (Figure 2G). To validate these findings *in vivo*, OIR mice received intraperitoneal lactate injections (P13-P16) to augment lactylation, with retinal collection at P17 33, 42-44 (experimental timeline, Figure 2H). Retinal histone lactylation was elevated in lactate-treated OIR mice relative to vehicle controls (Figure 2I). Costaining of histone lactylation with IsoB4, an endothelial marker, demonstrated the specific enrichment of H3K18la in the pathological neovascular tufts of lactate-treated OIR retinas (Figure 2J). Retinal flat-mount immunofluorescence staining confirmed the increased pathological neovascularization in lactate-treated OIR mice at P17 (Figure 2K). These findings indicate that elevated histone lactylation directly enhanced the angiogenic capacity of vascular endothelia.

### Depression of H3K18 lactylation rescues ischemia-driven retinal neovascularization

**Figure 3:**
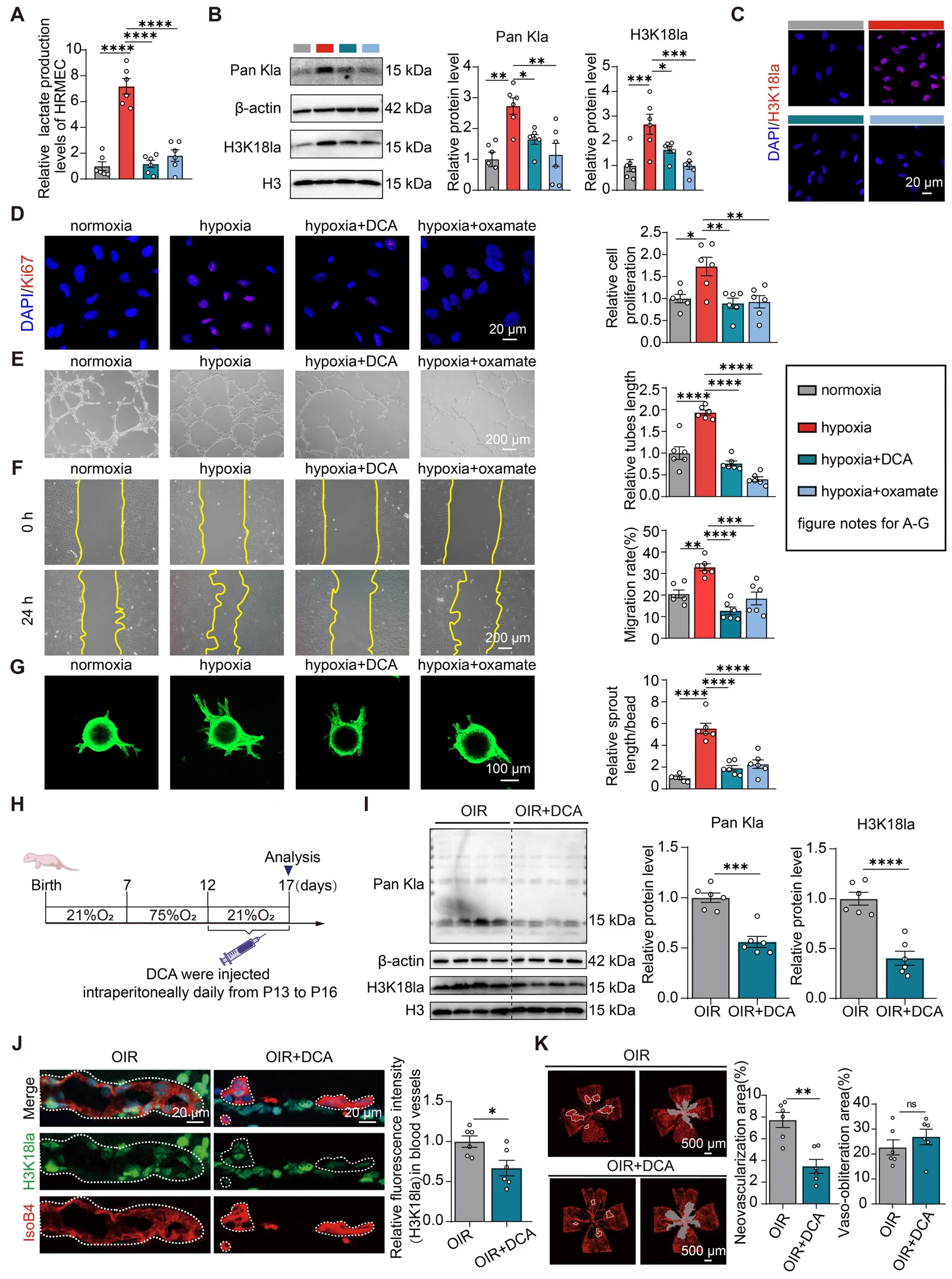
** A decrease in H3K18la levels leads to a reduction in retinal neovascularization. (A)** Intracellular lactate dynamics in HRMECs under hypoxia following treatment with the lactate inhibitors oxamate (20 mM) and DCA (20 mM), quantified using a colorimetric assay (n = 6). **(B)** Immunoblots for pan-Kla and H3K18la in HRMECs after 24 h of treatment with lactate inhibitors (n = 6). **(C)** Representative immunofluorescence images showing H3K18la localization in inhibitor-treated HRMECs (24 h). Scale bar: 20 µm (n = 6). **(D-G)** Functional assays in HRMECs exposed to hypoxia and lactate inhibitors for 24 h: (D) Ki67 immunofluorescence staining (proliferation), (E) tube formation, (F) scratch wound healing, and (G) spheroid sprouting assays (n = 6 each). **(H)** Schematic representation of OIR modeling and DCA (200 mg/kg) dosing via daily intraperitoneal injection (P13–P16). **(I)** Quantitative western blot analysis of pan-Kla and H3K18la in the retinas of DCA-treated OIR mice (n = 6/group). **(J)** Immunofluorescence analysis revealed suppression of H3K18la in the retinal vasculature of DCA-treated OIR mice versus vehicle controls. Scale bar: 20 µm (n = 6). **(K)** Retinal whole-mounts after saline or DCA intraperitoneal injection with quantification of neovascular and avascular zones. Scale bar: 500 µm (n = 6 each). Statistical analysis was performed using a one-way ANOVA with Tukey’s multiple comparison test (A-G) or an unpaired two-tailed Student’s *t*-test (I-K). Data presented as mean ± SEM (*P < 0.05, **P < 0.01, ***P < 0.001, ****P < 0.0001).

To establish the causal role of lactylation in pathological angiogenesis, we employed two mechanistically distinct lactate inhibitors: DCA (a PDK1 inhibitor that redirects pyruvate toward acetyl-CoA) and sodium oxamate (an LDHA inhibitor that blocks pyruvate-to-lactate conversion) 45, 46 (metabolic mechanism schema, Figure S3A). In hypoxic HRMECs, both inhibitors significantly attenuated lactate accumulation (Figure 3A) and suppressed hypoxia-induced hyperlactylation, as confirmed by western blotting (Figure 3B) and immunofluorescence staining (Figure 3C, and S3B). Functional profiling demonstrated that lactylation inhibition markedly reduced the angiogenic capacity, as evidenced by diminished proliferation (Ki67 immunofluorescence, Figure 3D), suppressed tube formation (Figure 3E), impaired migration (Figure 3F), and spheroid sprouting (Figure 3G).

*In vivo* validation using intraperitoneal DCA administration in OIR mice (P13-P16; experimental timeline Figure 3H) showed reduced retinal histone lactylation compared with that in vehicle controls (Figure 3I-J). Retinal flat-mount analysis confirmed significant attenuation of pathological neovascular tufts in DCA-treated mice (Figure 3K). Combined genetic and pharmacological evidence indicate that lactylation contributed significantly to the regulation of pathological retinal neovascularization.

### H3K18 lactylation gates pathological *ETS1* activation in retinal ECs

**Figure 4:**
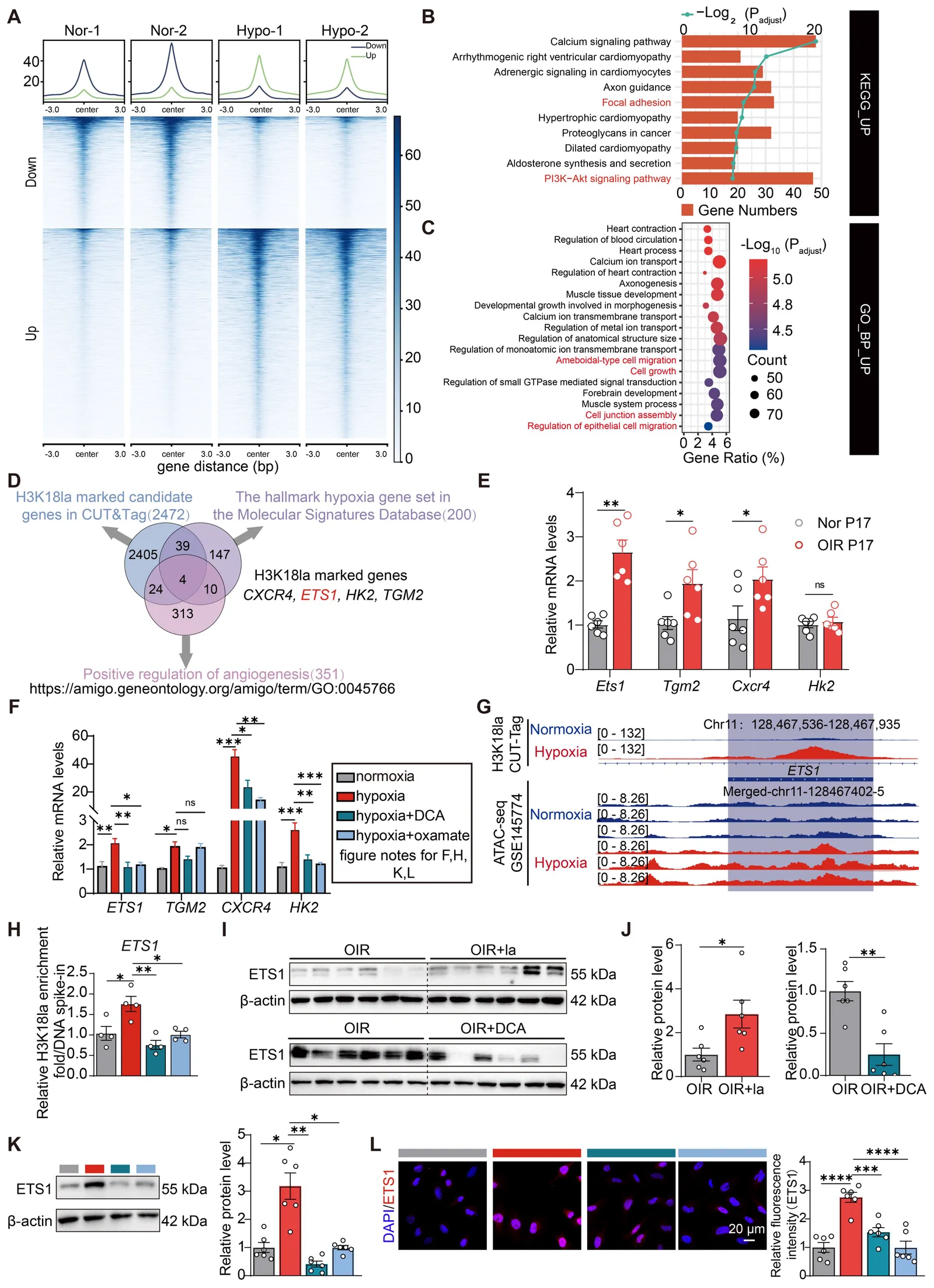
** H3K18 lactylation in endothelial cell regulates the transcription of ETS1. (A)** Metaplots of H3K18la CUT&Tag read density relative to transcription start sites (TSS). Additionally, heatmaps of binding peaks in HRMECs were displayed across normoxic and hypoxic environments. Color intensity reflects read density. **(B)** KEGG biological pathway enrichment analysis of genes associated with hypoxia-induced H3K18la peaks (FDR-adjusted *P* < 0.05). **(C)** Gene ontology (GO) enrichment bubble chart for genes harboring hypoxia-upregulated H3K18la peaks. **(D)** Integrative analysis of CUT&Tag and RNA-seq identifies four H3K18la-regulated target genes. **(E)** RT-qPCR validation of target mRNAs in retinas from OIR and control mice (n = 6). **(F)** RT-qPCR analysis of target gene expression in hypoxic HRMECs treated with or without glycolytic inhibitors (DCA and oxamate) (n = 6). **(G)** Integrative Genomics Viewer (IGV) tracks showing H3K18la CUT&Tag signals at the *ETS1* locus compared to published ATAC-seq data in hypoxic HUVEC. The highlighted region denotes the H3K18la peak overlapping with the *ETS1* enhancer (Chr11:128, 467, 536–128, 467, 935). **(H)** H3K18la occupancy at *ETS1* was determined by CUT&RUN–qPCR in hypoxic HRMECs with or without glycolytic inhibitors (n = 4). **(I,J)** Western blot (I) and quantification (J) of ETS1 protein levels in retinas from OIR mice treated with lactate or DCA (200 mg/kg) versus saline controls (n = 6/group). **(K)** Glycolytic inhibitors attenuate ETS1 protein induction in hypoxic HRMECs (n = 6). **(L)** Representative immunofluorescence images of ETS1 (red) and DAPI (blue) in HRMECs under hypoxia, with or without glycolytic inhibitors. Scale bar: 20 µm (n = 6). Data were analyzed using a one-way ANOVA with Tukey’s multiple comparison test (F,H,K,L) or a two-tailed unpaired Student’s *t*-test (E,J). Bars/points indicate mean ± SEM. Significance: *P < 0.05, **P < 0.01, ***P < 0.001, ****P < 0.0001.

Histone lactylation modulates gene transcription via epigenetic mechanisms. To assess the involvement of histone lactylation in influencing the pro-angiogenic gene expression, we performed H3K18la-specific CUT&Tag 47 in hypoxic HRMECs. Using a validated H3K18la antibody (ChIP-grade), we mapped the genome-wide occupancy in hypoxic HRMECs and characterized the distribution patterns, regulated genes, associated pathways, and transcription factors (Figure S4A). H3K18la CUT&Tag heatmaps revealed altered genomic occupancy (± 3kb from transcription start sites [TSS]), with hypoxia inducing 2,472 upregulated and 1,940 downregulated genes (Figure 4A). Peaks were predominantly enriched in the enhancer regions (Figure S4B), and pathway enrichment analysis of H3K18la-marked upregulated genes revealed significant involvement in cell adhesion, migration, and junction assembly (Figure 4B-C).

Next, we elucidated the mechanism by which histone lactylation regulates vascular gene expression under hypoxic conditions. By overlapping the gene sets among the H3K18la CUT&Tag data, hypoxia-responsive genes 48, and pro-angiogenic Gene ontology (GO) terms (GO: 0045766), four lactate-regulated functional targets were identified: *CXCR4*, *ETS1*, *HK2*, and *TGM2* (Figure 4D and S4C). RT-qPCR analysis confirmed *Ets1* upregulation in OIR tissues (Figure 4E), whereas hypoxia increased *ETS1* expression in HRMECs and lactate inhibition (DCA/oxamate) reversed this effect (Figure 4F). Concordantly, the publicly available ATAC-seq data 49 demonstrated enhanced chromatin accessibility at the *ETS1* locus in HUVECs after hypoxia (Figure 4G). CUT&RUN–qPCR validation revealed the co-localization of H3K18la and H3K27ac 50 at the *ETS1* locus under hypoxia (Figure S4D-E). Hypoxic stimulation significantly increased H3K18la occupancy at the *ETS1* genomic locus, whereas pharmacological inhibition of glycolysis attenuated this enrichment (Figure 4H). Collectively, hypoxia-enhanced glycolytic flux promoted lactate-dependent H3K18la modification, driving *ETS1* transcriptional activation.

Western blotting confirmed ETS1 upregulation in OIR retinas (Figure S4F), and immunofluorescence staining revealed that the increased ETS1 expression colocalized with the vasculature (Figure S4G). Consistent with this, ETS1 protein levels increased in lactate-treated OIR mice but decreased with DCA treatment, as shown by immunoblotting (Figure 4I-J) and immunofluorescence staining (Figure S4H-I).

In HRMECs, hypoxia-induced ETS1 upregulation was attenuated by lactate inhibitors (DCA/oxamate), as shown by western blotting and immunofluorescence staining (Figure 4K-L). Mechanistically, H3K18la serves as an epigenetic regulator that promotes the transcription of pro-angiogenic genes, particularly *ETS1*, suggesting that this modification is a pivotal driver of pathological neovascularization.

### H3K18la drives pathological angiogenesis in retinopathy through *ETS1* transactivation

**Figure 5:**
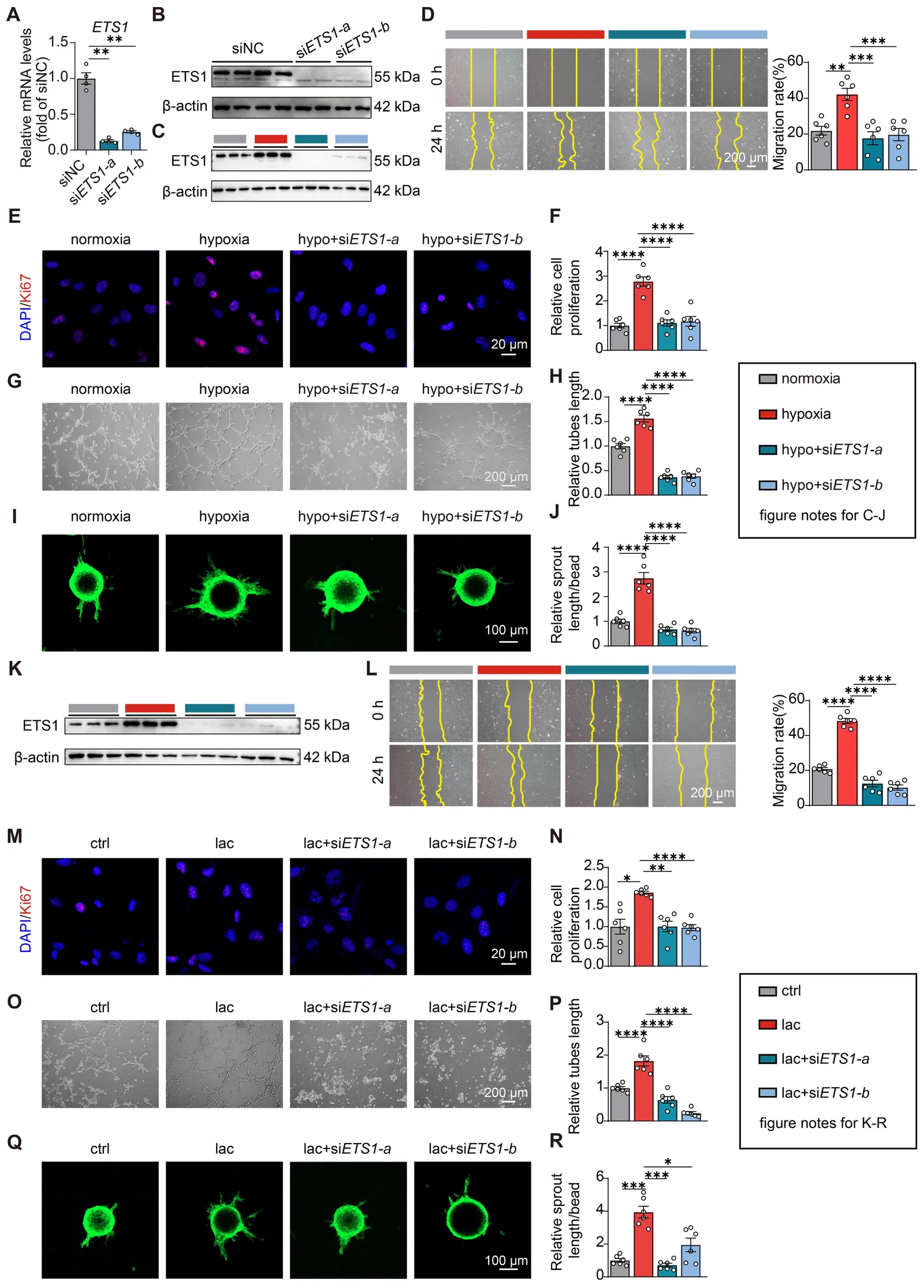
** H3K18la drives pathological angiogenesis in retinopathy through ETS1 transactivation. (A,B)** *ETS1* knockdown efficiency in HRMECs transfected with *ETS1* siRNA (50 nM) vs. scrambled control (NC siRNA) as validated by RT-qPCR (A) and western blotting (B) after 24 h. **(C)** Representative immunoblots of ETS1 in hypoxic HRMECs following *ETS1*-knockdown (NC siRNA/*ETS1* siRNA group) (n = 6). **(D-J)** Functional validation of *ETS1*-knockdown in hypoxic HRMECs:- D: Scratch wound healing assay (representative images, scale bar: 200 μm);- E,F: Ki67 immunofluorescence staining (E: representative images; F: quantification. Scale bar: 20 μm);- G,H: Tube formation (G: representative images; H: quantification. Scale bar: 200 μm);- I,J: Spheroid sprouting (I: representative images; J: quantification). Scale bar: 100 μm)(All n = 6). **(K)** Immunoblotting confirming that *ETS1* knockdown attenuates lactate-induced upregulation of ETS1 expression. **(L-R)** Phenotypic rescue assays under lactate stimulation (10 mM):- L: Scratch wound healing assay (Scale bar: 200 μm);- M,N: Ki67 immunofluorescence staining (M: images; N: quant. Scale bar: 20 μm);- O,P: tube formation (O: images; P: quant. Scale bar: 200 μm); - Q,R: spheroid sprouting (Q: images; R: quant. Scale bar: 100 μm)(All n = 6). Statistical analysis: One-way ANOVA with Tukey’s multiple comparison test. Data presented as mean ± SEM (*P < 0.05, **P < 0.01, ***P < 0.001, **P < 0.0001).

To define the functional contribution of *ETS1* to H3K18la-driven angiogenesis, we performed *ETS1* knockdown using siRNA 51. RT-qPCR and immunoblotting confirmed a significant reduction at both the transcriptional and translational levels (Figure 5A-B). Knocking down *ETS1* attenuated the hypoxia-induced ETS1 upregulation (Figure 5C). Functional angiogenesis assays showed that hypoxia significantly enhanced HRMEC migration and proliferation, whereas *ETS1* knockdown abrogated these effects (Figure 5D-J). Moreover, *ETS1* depletion reversed lactate-induced ETS1 elevation (Figure 5K) and suppressed lactate-enhanced migratory and proliferative responses (Figure 5L-R).

### PFKFB3 fuels metabolic reprogramming to drive lactate-dependent histone lactylation

**Figure 6:**
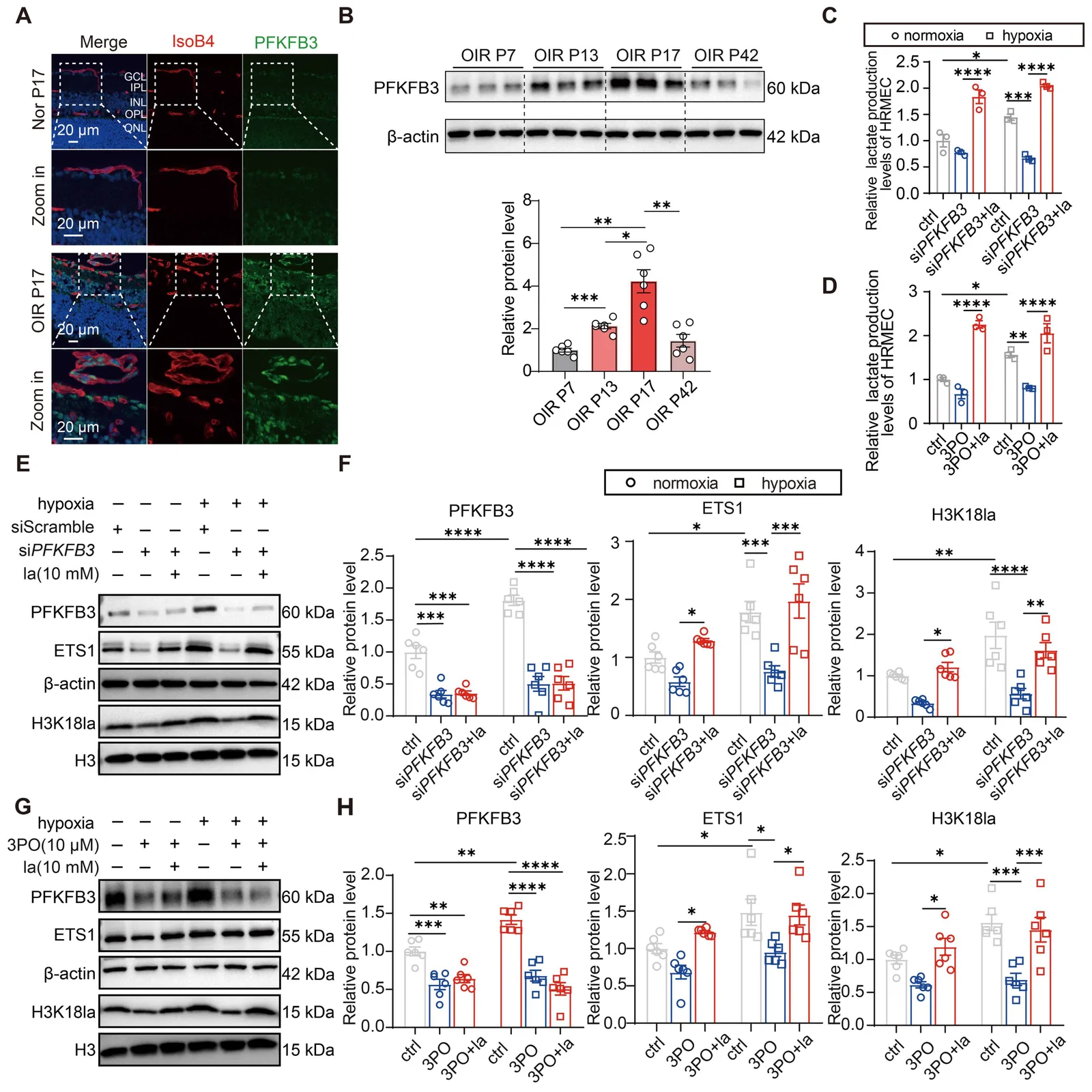
** PFKFB3 increases lactate generation, leading to histone lactylation. (A)** Immunofluorescence analysis of PFKFB3 in retinal sections from normoxic (Nor P17) and oxygen-induced retinopathy (OIR P17) mice. Scale bar: 20 μm (n = 6). **(B)** Immunoblotting (top) and densitometric quantification (bottom) of retinal PFKFB3 in OIR mice and OIR P7 mice (n = 6/group). β-actin loading control shown. **(C)** Intracellular lactate levels in hypoxic HRMECs with *PFKFB3*-knockdown (si*PFKFB3*), with or without exogenous lactate stimulation (10 mM). Control: scrambled siRNA (n = 3). **(D)** Lactate dynamics in normoxic/hypoxic HRMECs treated with PFKFB3 inhibitor 3PO (10 μM) with or without exogenous lactate (10 mM) (n = 3). **(E,F)** Immunoblot analysis of targets in si*PFKFB3*-transfected HRMECs with or without exogenous lactate (10 mM) under normoxia/hypoxia (n = 6). **(G,H)** Western blots of targets in 3PO-treated (10 μM) HRMECs with or without exogenous lactate stimulation under normoxia/hypoxia (n = 6). **(B)** One-way ANOVA with Tukey’s multiple comparison test; **(C-H)** two-way ANOVA with Tukey’s multiple comparison test. Data are presented as mean ± SEM. Significance: *P < 0.05, **P < 0.01, ***P < 0.001, ****P < 0.0001.

Endothelial glycolysis is the dominant metabolic pathway that sustains angiogenesis. Next, we investigated the key glycolytic enzymes that amplify this flux. Published RNA-seq data 52 revealed significant upregulation of *Pfkfb3*, *Pdk1*, and *Ldha* in OIR retinas (Figure S5A), as confirmed by RT-qPCR (Figure S5B). As a metabolic enzyme that produces fructose-2,6-bisphosphate (F2,6BP), PFKFB3 allosterically stimulates the essential glycolytic enzyme PFK-1 Consistent with previous reports 11, 53, PFKFB3 expression was upregulated in OIR mice and in hypoxic endothelia (Figure 6A, S5C-D), peaking at P17 (Figure 6B). Thus, PFKFB3 has emerged as an important glycolytic regulator of endothelial energy homeostasis and angiogenic activation.

In hypoxic endothelia, *PFKFB3* knockdown or pharmacological inhibition (3PO) significantly reduced lactate production, whereas sodium lactate reversed this effect (Figure 6C-D). Immunoblotting confirmed that impaired PFKFB3 function attenuated hypoxia-induced H3K18la expression, and lactate addition restored this modification, while partially reversing ETS1 upregulation (Figure 6E-H).

### P300-mediated H3K18 lactylation epigenetically drives pathological retinal neovascularization

P300 functions as a lactate-writing enzyme that catalyzes protein lactylation by utilizing lactyl-CoA to transfer lactyl groups to specific lysine residues, thereby establishing PTM 17, 54. Elevated P300 expression was observed in OIR retinas and hypoxic endothelia (Figure S6A-B). Pharmacological inhibition with C646 attenuated lactate/hypoxia-induced H3K18la elevation in HRMECs 55, 56 (Figure S6C). Genetic ablation via si-*P300* reduced H3K18la levels 57 (Figure S6D). H3K18la physically interacted with P300, as demonstrated by Co-IP (Figure S6E), and nuclear colocalization was confirmed (Figure S6F). Crucially, P300 silencing diminished hypoxia-enhanced H3K18la occupancy at the *ETS1* locus (Figure S6G) and concurrently impaired angiogenesis-associated functions (Figure S6H-M).

### ETS1 orchestrates ischemia-driven pathological neovascularization *in vivo*

**Figure 7:**
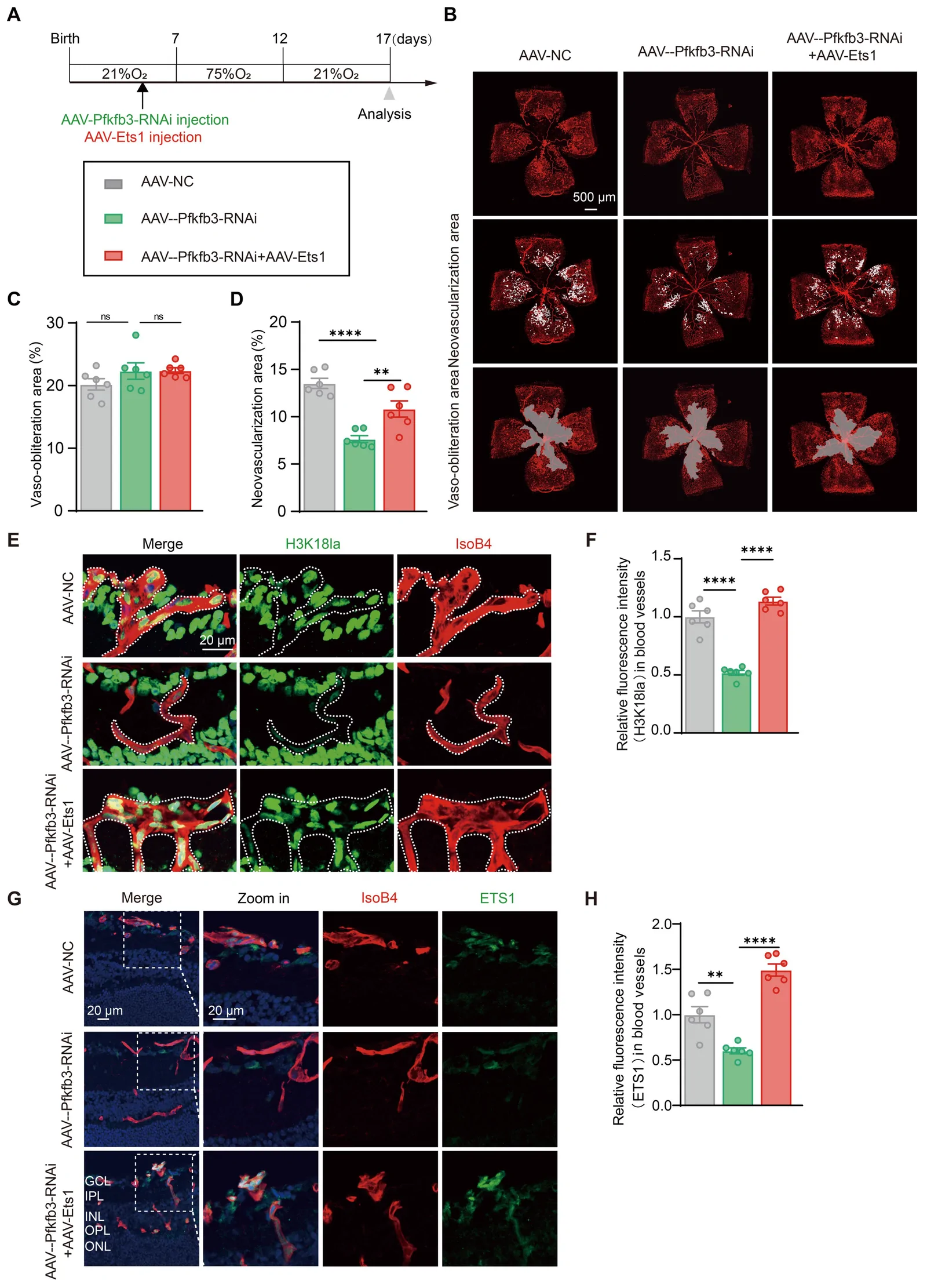
** ETS1 promotes pathological retinal angiogenesis *in vivo.* (A)** Schematic representation of genetic interventions in a murine oxygen-induced retinopathy (OIR) model via retro-orbital injection of AAV-Pfkfb3-RNAi and AAV-Ets1. **(B)** Representative retinal whole-mounts from OIR P17 mice with specific genotypes stained with IsoB4. Vasobliteration (VO, gray) and pathological neovascularization (NV, white) are highlighted. Scale bar: 500 μm. **(C,D)** Quantification of the VO area (C) and pathological NV area (D) as a percentage of the total retinal area (n = 6). **(E)** Immunofluorescence analysis of retinal cross sections showing H3K18la expression in the retinal vasculature across the three experimental groups at P17. Scale bar: 20 μm. **(F)** Quantification of H3K18la fluorescence intensity within the IsoB4-positive vasculature across groups (n = 6). **(G,H)** Representative immunofluorescence images (G) and quantification (H) of ETS1 protein expression in the retinal vasculature across the three OIR groups at P17. Scale bar: 20 μm (G), n = 6 (H). Statistical analysis: One-way ANOVA with Tukey’s multiple comparison test. Data represent mean ± SEM. **P < 0.01, ****P < 0.0001.

Functionally validated *in vivo*, the PFKFB3-lactylation-ETS1 axis was investigated using adeno-associated virus (AAV)-mediated delivery of Pfkfb3-RNAi or Ets1 into OIR models. On P3, the retro-orbital injection of control (AAV-NC), AAV-Pfkfb3-RNAi, or AAV-Ets1 enabled angiogenic phenotyping 58 (Figure 7A). Compared to AAV-NC controls, *Pfkfb3*-knockdown retinas exhibited reduced neovascular area (Figure 7B-D) and attenuated H3K18la immunoreactivity in the endothelial zones (Figure 7E-F), while concomitant *Ets1* overexpression reversed both the neovascular area and H3K18la level. Immunofluorescence quantification revealed diminished ETS1 and PFKFB3 signals in knockdown retinas;* Ets1* addback restored ETS1 expression but not PFKFB3 expression (Figure 7G-H, S7A-B).

Consistent with our observations, glycolytic suppression reduced histone lactylation, ultimately mitigating pathological angiogenesis. Conversely, an enhanced glycolytic flux exacerbated these pathological manifestations. Collectively, the PFKFB3-driven glycolytic flux orchestrates histone lactylation, which critically potentiates the pathogenesis of ischemic retinopathy.

## Discussion

Our study elucidates the glycolytic-lactate-histone lactylation axis, which epigenetically participates in pathological angiogenesis in ischemic retinopathy. Specifically, hypoxic microenvironments upregulate PFKFB3 to enhance the glycolytic flux and lactate production, thereby inducing H3K18la. This modification preferentially occupies the enhancer regions to transactivate ETS1 expression, which orchestrates endothelial cell proliferation, migration, and tube formation, culminating in aberrant retinal neovascularization. Mechanistically, histone acetyltransferase P300 functions as a putative “writer enzyme” catalyzing H3K18la 17, 57, 59. Pharmacological lactate inhibition significantly reduced H3K18la levels and ETS1 expression, thereby suppressing pathological neovascularization in animal models (graphical abstract). This study integrated metabolic reprogramming, epigenetic modulation, and angiogenic signaling. The study also revealed that lactate/lactylation elevation is a component of a broader metabolic-epigenetic axis that operates within the context of retinal ischemia.

Vascular ECs, which constitute the innermost layer of blood vessels, are directly exposed to the bloodstream. This permits acute sensing and response to fluctuations in circulatory nutrient concentrations. Recent studies have indicated that metabolic reprogramming is a key mechanism that drives angiogenesis. ECs exhibit unique metabolic profiles, characterized by a high dependence on glycolysis for glucose metabolism. Their glucose uptake and consumption rates are similar to those of malignant tumor cells. Multiple studies have established that key glycolytic regulators, including *PFKFB3*, *ADORA2A*, and *HK2*, play pivotal roles in maintaining endothelial energy homeostasis and regulating angiogenesis 11-13. Under pathological conditions, such as cancer or diabetes, ECs undergo adaptive metabolic alterations that frequently lead to functional impairment. In addition to glucose metabolism, ECs display distinctive patterns of lipid and amino acid metabolism. For instance, inhibiting CPT1A, a key enzyme in fatty acid oxidation in ECs, acts without compromising energy metabolism or redox balance, yet impairs *de novo* nucleotide synthesis, consequently disrupting DNA replication 60. Inhibiting PHGDH, the key enzyme in the serine synthesis pathway, impairs heme synthesis, reduces purine and pyrimidine production, and causes mitochondrial respiration defects and oxidative stress.^61^. Collectively, metabolic signaling is not only essential for maintaining normal endothelial function but also orchestrates all stages of angiogenesis through diverse pathways. This emphasizes the importance of endothelial metabolism as a therapeutic target, demonstrating its potential for clinical application in angiogenesis-related disorders.

The “Retinal Warburg Effect” occurs in the vertebrate retina and is characterized predominantly by aerobic glycolysis during glucose metabolism. Consequently, lactate accumulation is associated with increased aerobic glycolysis and lactate production, particularly during retinal angiogenesis. Lactate levels were significantly elevated in the blood of infants with ROP compared with those in non-ROP controls 44. In OIR mouse retinal lysates, 41 lactate metabolism-associated genes were differentially expressed 62, potentially driving ROP pathogenesis by modulating lactate metabolism to alter immune and metabolic processes. Plasma lactate and citrate levels are correlated with microvascular damage in the macular and optic nerve regions 63. Consistent with these findings, retinal lactate levels were higher in OIR mice than those in control mice. Alterations in the metabolic microenvironment can directly regulate gene expression via epigenetic mechanisms. Under hypoxic conditions, ECs predominantly use glycolysis to generate substantial lactate 64. Beyond its metabolic role, lactate acts as a signaling molecule that influences the chromatin state and transcriptional activity via histone lactylation modifications 17, 22. The lactylation of H3K18la is a common post-translational modification. This involves a covalent lactyl group linkage to the flexible H3 tail residue, which modulates chromatin architecture and gene transcription to affect cellular processes and disease pathogenesis 50, 65. Our findings indicate that H3K18la is the predominant histone modification under both *in vivo* ischemic retinopathy and *in vitro* hypoxic conditions. Collectively, these findings provide novel insights into the vascular pathologies associated with metabolic dysregulation.

Lactate metabolism, particularly lactylation, is pivotal in the modulation of retinal homeostasis. DR, a primary cause of permanent vision loss among adults of working age, is characterized by increased histone lactylation during the proliferative phase. This modification markedly upregulates the expression of m6A demethylases, including fat mass and obesity-associated protein (FTO). Augmented FTO expression accelerates EC cycling by regulating CDK2 mRNA stability in a YTHDF2-dependent manner 66. In ECs, the feedback loop between H3K9 lactylation (H3K9la) and histone deacetylase 2 (HDAC2) drives VEGF-induced angiogenesis. VEGF stimulation triggers H3K9la upregulation in ECs, whereas pharmacological glycolysis inhibition reduces H3K9la levels and attenuates neovascularization 67. In this study, pan-lysine lactylation (pan- Kla) and H3K18la modifications were elevated in P17 OIR mice compared with those in normoxic controls. Integrated CUT & Tag for H3K18la and RNA-seq analyses, combined with pro-angiogenic gene datasets, identified four hypoxia-responsive angiogenic regulators: *CXCR4*, *ETS1*,* HK2*, and *TGM2*. Subsequent validation using RT-qPCR in animal and cellular models confirmed *ETS1* as the primary H3K18la-regulated downstream target. Its expression is directly regulated by H3K18la and strongly correlates with hypoxic responses and pro-angiogenic phenotypes. Functional analyses demonstrated that *ETS1* silencing significantly impaired endothelial cell proliferation and migration and reversed the pro-angiogenic effects induced by lactate or hypoxia, confirming the essential role of ETS1 in this regulatory axis. Over the past decade, the Ets1 transcription factor has been established as a critical regulator of tumor invasion, cardiac development, and myocardial survival within the ETS family 68-70. Intriguingly, AAV-mediated overexpression of ETS1 unexpectedly elevated H3K18la levels, which is inconsistent with previous results. Multiple studies have demonstrated that Ets1 promotes glycolytic metabolism (Warburg effect) while suppressing oxidative phosphorylation (OXPHOS): Ets1 protects against cardiac ischemia/reperfusion injury via hexokinase activation-enhanced glycolysis 71; Ets1 overexpression reduces mitochondrial load, and impairs ATP synthase (Complex V) function by inducing dynamin-related protein 1 (Drp1), thereby shifting cellular metabolism toward glycolysis 72. These results indicate a possible self-reinforcing cycle linking ETS1 and glycolytic metabolism; however, the precise mechanisms warrant further investigation.

In epigenetic regulation, “writer” enzymes catalyze specific modifications on substrate molecules to establish functional epigenetic marks. Histone lactylation induces local conformational changes, loosening DNA-histone interactions which facilitate “reader” protein recognition and downstream transcriptional regulation. The pivotal discovery that P300 functions as a lactylation writer occurred in 2019 when Zhang et al. first proposed acetyltransferase P300 as a mediator of histone lysine lactylation through its acetyltransferase structural domain. Using lactyl-CoA as the lactyl donor, P300 catalyzes lactylation and exhibits remarkable catalytic flexibility, capable of transferring multiple types of acyl groups to lysine residues. Thus, the shared regulatory machinery suggests potential competitive regulation between lactylation and acetylation. However, they showed that histone lactylation increased under hypoxic or lactate conditions, whereas acetylation remained stable. Through functional validation, we demonstrated that P300 is the primary lactyltransferase in HRMECs. P300 inhibition via siRNA or C646 attenuated histone lactylation, consequently suppressing hypoxia- and lactate-induced proliferation and migration of HRMECs 73. We note: (i) P300 may not be the exclusive lactyltransferase; (ii) its pleiotropic functions in catalyzing multiple acylations (acetylation/crotonylation/β-hydroxybutyrylation) 74-76 could introduce confounding effects; and (iii) contributions of other P300-mediated pathway to pathological angiogenesis cannot be excluded 73, 77, 78. Collectively, this study demonstrates the P300-mediated lactylation capacity; however, we cannot exclude that P300-mediated additional acylations may contribute to the angiogenesis phenotypes, and further investigations are necessary.

Since its formal discovery in 2019, lysine lactylation has remained a nascent research field. Fundamental mechanistic questions remain unaddressed, and these limitations persist in our current study. Global manipulation of lactate levels, whether through exogenous supplementation, glycolytic inhibitors, or metabolic enzyme modulation, inevitably alters lactylation patterns across multiple sites. Future studies should focus on developing precise pharmacological agents capable of targeting specific sites. Additionally, the generation of point mutation models would provide definitive evidence to establish a causal relationship between modifications at this specific site and the observed phenotypes. The potential toxicity and off-target effects at high drug concentrations were not fully excluded. In addition to histones, advances in lactyl proteomics have led to extensive investigations into non-histone lysine lactylation (Kla). Elevated lactate levels induce histone lactylation, which subsequently remodels chromatin architecture and reprograms gene expression. Non-histone lactylation modulates protein function by inducing the conformational rearrangement of target proteins. Accumulating evidence confirms the regulatory breadth of Kla beyond that of histones 44, 54, 79. However, this study did not characterize non-histone lactylation events. Future studies should delineate the broader regulatory networks orchestrated by non-histone lactylation.

## Conclusion

From a translational perspective, our findings identified druggable targets within the metabolic-epigenetic axis. Although widely used, current anti-VEGF therapies have limitations including therapeutic resistance and tissue nonselectivity. Current Kla modulation primarily targets lactate metabolism, with MCT1/4 transporters (key lactate shuttles) showing promise in psoriasis, neuroinflammation, and ocular disorders 80-83. The LDH inhibitor GNE-140 attenuated PM2.5-induced pulmonary inflammation and fibrosis in mice by suppressing glycolysis and consequent histone Kla 84. Our intervention, targeting PFKFB3 (a rate-limiting controller of glycolytic flux) and histone lactylation, offers viable alternative/synergistic therapeutics for pathological angiogenesis with strong translational potential.

Collectively, we uncovered a PFKFB3-lactate-H3K18la-ETS1 signaling axis that contributes to pathological angiogenesis in ischemic retinopathy through metabolic-epigenetic crosstalk. This pathway not only reveals a new regulatory tier in vascular biology but also offers novel therapeutic targets and conceptual frameworks for ocular neovascular diseases.

## Supplementary Material

Supplementary figures and tables.

## Abbreviations

PDR: proliferative diabetic retinopathy
AMD: age-related macular degeneration
ROP: retinopathy of prematurity
RVO: retinal vein occlusion
VEGF: vascular endothelial growth factor
ECs: endothelial cells
MCTs: monocarboxylate transporters
PTM: post-translational modification
LPS: lipopolysaccharide
VSMC: vascular smooth muscle cell
SASP: senescence-associated secretory phenotype
MAC: medial arterial calcification
H3K18la: histone H3 lysine 18 lactylation
PAH: pulmonary arterial hypertension
PDK: pyruvate dehydrogenase kinase
OIR: oxygen-induced retinopathy
ECM: endothelial cell medium
DCA: sodium dichloroacetate
PFA: paraformaldehyde
GO-BP: Gene Ontology Biological Process
DR: diabetic retinopathy
HRMECs: human retinal microvascular endothelial cells
FTO: fat mass and obesity-associated protein
HDAC: histone deacetylase
IGV: Integrative Genomics Viewer
IsoB4: Isolectin B4
