# Supplementary material for: Lactylation at H3K18 drives pathological angiogenesis via metabolic-epigenetic crosstalk in ischemic retinopathy
Source: Theranostics. 2026 Aug 12;16(15):8650–68. doi: 10.7150/thno.136054 (PMC13549230; doi:10.7150/thno.136054)
Supplement: Supplementary file 1 [file thnov16p8650s1.pdf]

Supplemental Materials

Lactylation at H3K18 drives pathological angiogenesis via metabolic-epigenetic crosstalk in ischemic retinopathy

Xin Chen, Yuhan Jiang, Weiqi Qian, Yi Lei, Siyue Chen, Yutian Zhang, Minghui Liang, Yuming Liu, Xiaohong Wang, Ding Ai, Xuyang Yao, Hua Yan

Data Online Supplement Figures S1-S7

Data Online Table S1-S4

Supplement Figures and Figure legends

Figure S1

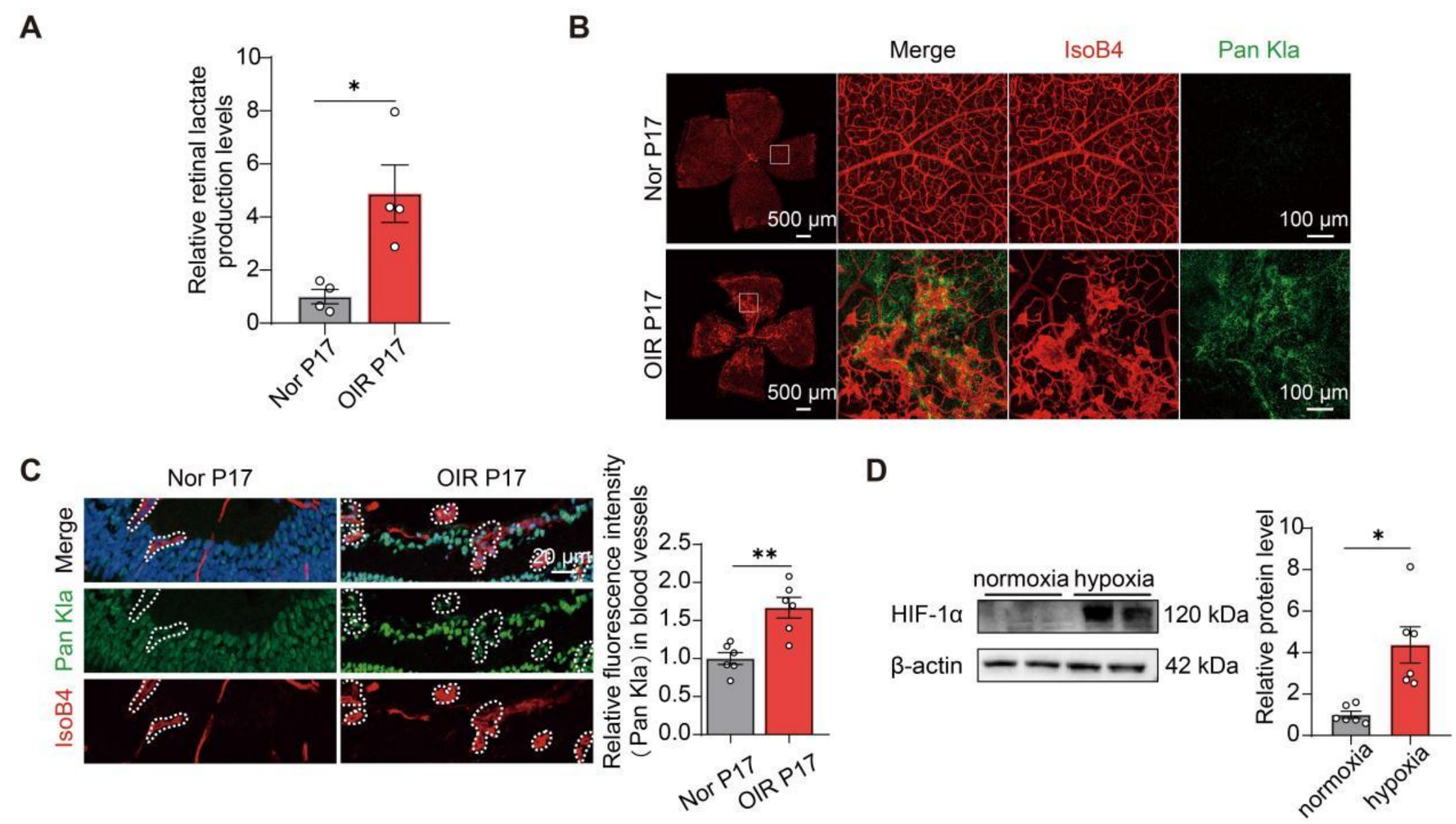

Figure S1. Elevated pan-lactylation levels exhibit significant association with retinal neovascularization in oxygen-induced retinopathy (OIR) models

A: Retinal lactate levels were significantly elevated in oxygen-induced retinopathy (OIR) mice on postnatal day 17 (P17) compared to normoxic controls (Nor P17) (n = 4).

B: Representative retinal whole-mounts from Nor P17 and OIR P17 mice showing costaining with IsoB4 (red) and pan anti-lactyl-lysine antibodies (pan-Kla, green). Scale bars: 500 μm (left panels); 100 μm (right panels).

C: Vascular networks in P17 retinal sections co-labeled with IsoB4 (red) and H3K18la (green) antibodies. Scale bar: 20 μm (n = 6).

D: HIF-1α protein expression was analyzed by western blotting using β-actin as a loading control (n = 6).

Data were analyzed using two-tailed unpaired Student's *t*-test. Data represent mean ± SEM. Significance: *$P < 0.05$, **$P < 0.01$.

Figure S2

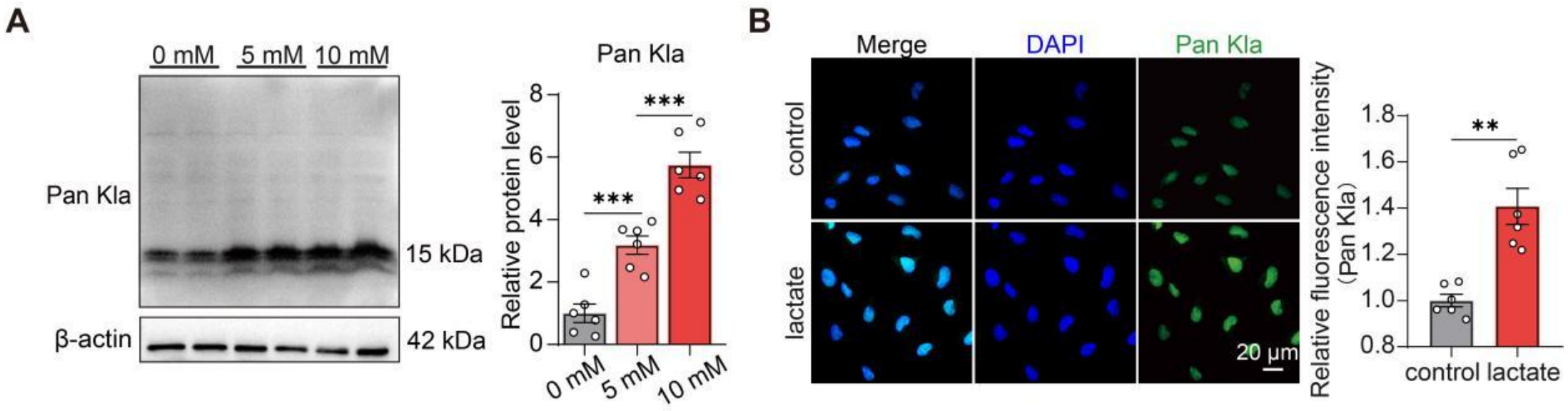
A
0 mM
5 mM
10 mM
Pan Kla
15 kDa
β-actin
42 kDa
Pan Kla
Relative protein level
8
6
4
2
0
***
***
0 mM
5 mM
10 mM
B
Merge
DAPI
Pan Kla
control
lactate
20 μm
Relative fluorescence intensity
(Pan Kla)
1.8
1.6
1.4
1.2
1.0
0.8
**
control
lactate

Figure S2. Lactate administration elevates global lactylation levels

A: HRMECs were treated with lactate (0, 5, and 10 mM) for 24 h. Lysates were immunoblotted with pan anti-lactyl-lysine antibody (pan-Kla), using β-actin as loading control (n = 6).

B: Immunofluorescence staining of HRMECs treated with or without 10 mM lactate, using the pan-Kla antibody (green) and nuclear counterstain DAPI (blue). Scale bar: 20 μm (n = 6).

Data were analyzed using a one-way ANOVA with Tukey's multiple comparison test (A) or a two-tailed unpaired Student's *t*-test (B). Data presented as mean ± SEM (**$P < 0.01$,***$P < 0.001$).

Figure S3

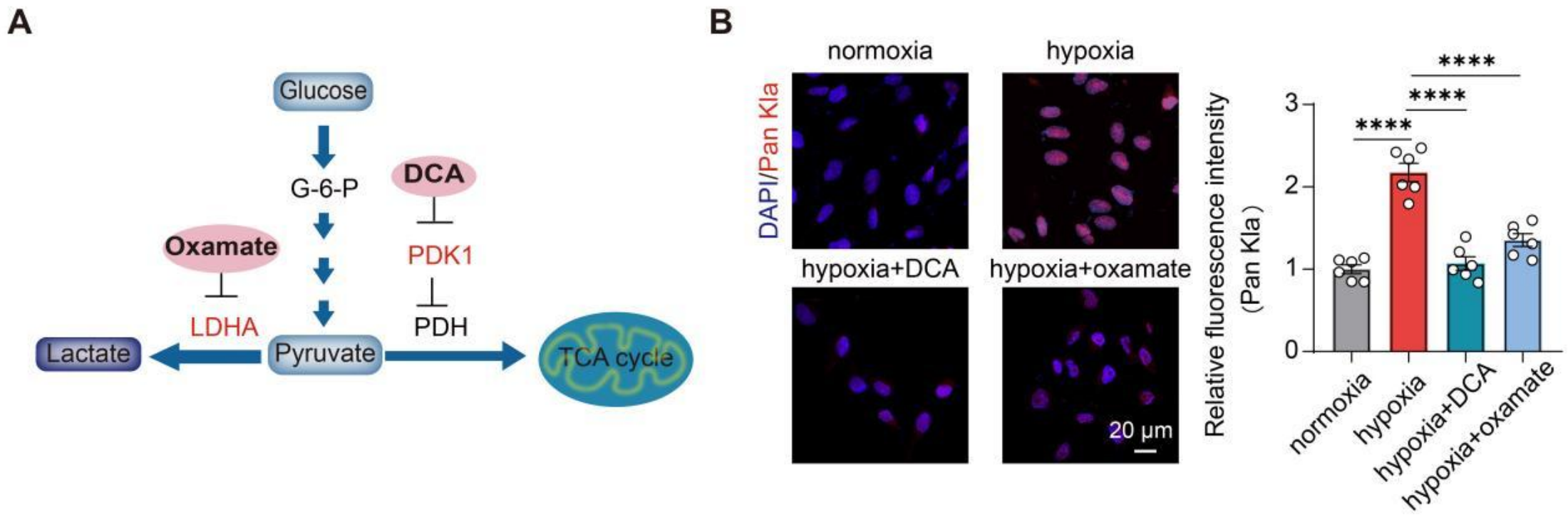
A
Glucose
G-6-P
DCA
Oxamate
PDK1
LDHA
PDH
Lactate
Pyruvate
TCA cycle
B
normoxia
hypoxia
hypoxia+DCA
hypoxia+oxamate
DAPI/Pan Kla
20 μm
Relative fluorescence intensity (Pan Kla)
****
****
****
0
1
2
3
normoxia
hypoxia
hypoxia+DCA
hypoxia+oxamate

Figure S3. Lactate inhibition reduces pan-lactylation levels

A: Dichloroacetate (DCA) activates pyruvate dehydrogenase (PDH) by inhibiting pyruvate dehydrogenase kinase 1 (PDK1), thereby shunting the glycolytic end product, pyruvate, into mitochondrial oxidative phosphorylation and reducing lactate production. Oxamate, a structural analog of pyruvate, functions as a competitive inhibitor of lactate dehydrogenase (LDHA). This inhibition suppresses lactate production from pyruvate, thereby disrupting the glycolytic flux.

B: HRMECs were exposed to (i) normoxia (21% O_2), (ii) hypoxia (1% O_2), (iii) hypoxia with 20 mM DCA, or (iv) hypoxia with 20 mM oxamate. Immunostaining with pan anti-lactyl-lysine antibody (pan-Kla, red) and nuclear counterstaining with DAPI (blue). Scale bar: 20 µm (n = 6).

Statistical analysis: One-way ANOVA with Tukey's multiple comparison test. Data are presented as the mean ± SEM (*****P* < 0.0001).

Figure S4

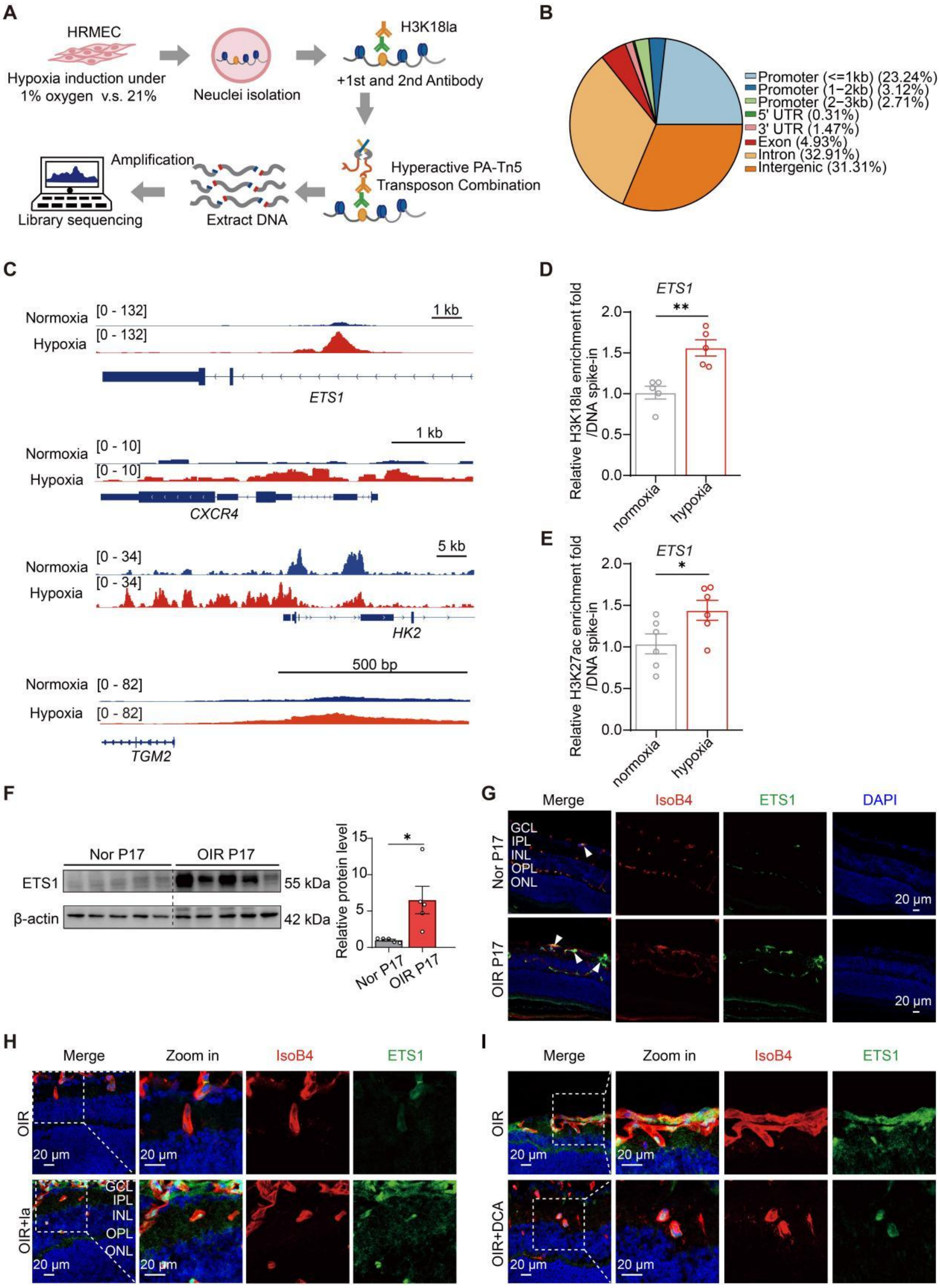
A
HRMEC
Hypoxia induction under
1% oxygen v.s. 21%
Neuclei isolation
H3K18la
+1st and 2nd Antibody
Hyperactive PA-Tn5
Transposon Combination
Extract DNA
Amplification
Library sequencing
B
Promoter (<=1kb) (23.24%)
Promoter (1−2kb) (3.12%)
Promoter (2−3kb) (2.71%)
5' UTR (0.31%)
3' UTR (1.47%)
Exon (4.93%)
Intron (32.91%)
Intergenic (31.31%)
C
Normoxia [0 - 132]
Hypoxia [0 - 132]
1 kb
ETS1
Normoxia [0 - 10]
Hypoxia [0 - 10]
1 kb
CXCR4
Normoxia [0 - 34]
Hypoxia [0 - 34]
5 kb
HK2
Normoxia [0 - 82]
Hypoxia [0 - 82]
500 bp
TGM2
D
ETS1
Relative H3K18la enrichment fold /DNA spike-in
normoxia
hypoxia
E
ETS1
Relative H3K27ac enrichment fold /DNA spike-in
normoxia
hypoxia
F
Nor P17
OIR P17
ETS1
55 kDa
β-actin
42 kDa
Relative protein level
Nor P17
OIR P17
G
Merge
IsoB4
ETS1
DAPI
Nor P17
OIR P17
GCL
IPL
INL
OPL
ONL
20 μm
H
Merge
Zoom in
IsoB4
ETS1
OIR
OIR+la
20 μm
I
Merge
Zoom in
IsoB4
ETS1
OIR
OIR+DCA
20 μm

Figure S4. H3K18 lactylation in endothelial cells may regulate *ETS1* transcription

A: Experimental workflow for CUT&Tag profiling of H3K18 lactylation in HRMECs under normoxic (21% O_2) and hypoxic (1% O_2) conditions.

B: H3K18la peaks demonstrate predominant enrichment in enhancer regions.

C: IGV visualization of H3K18la occupancy at *CXCR4*, *ETS1*, *HK2*, and *TGM2* loci.

D,E: Quantitative CUT&RUN-qPCR confirms the hypoxia-induced co-occupancy of H3K18la (n = 5) and H3K27ac (n = 6) at *ETS1* enhancer (Chr11:128, 467, 536–128, 467, 935).

F: Western blot analysis confirming ETS1upregulation in OIR retinas (n = 5).

G: Immunofluorescence analysis revealed enhanced ETS1 expression colocalized with the IsoB4 vasculature. Scale bar: 20 µm.

H,I: Immunofluorescence quantification reveals elevated ETS1 protein expression in sodium lactate-treated OIR mice (200 mg/kg), whereas dichloroacetate (DCA, 200 mg/kg) attenuated the expression compared with OIR. Scale bar: 20 µm.

Data were analyzed using two-tailed unpaired Student's *t*-test. Data represent mean ± SEM. Significance: *$P < 0.05$, **$P < 0.01$.

Figure S5

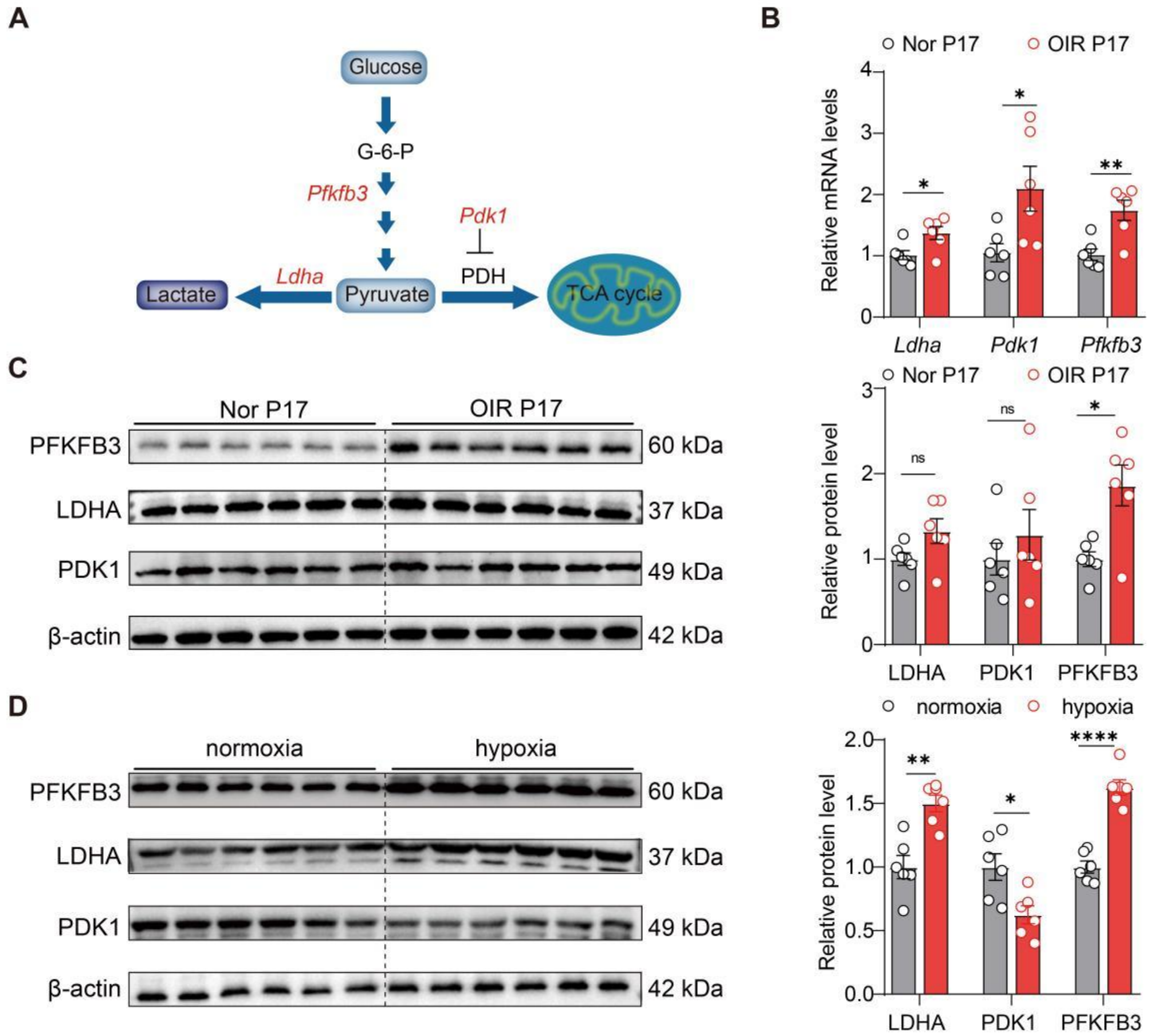
A
Glucose
G-6-P
Pfkfb3
Pdk1
PDH
Ldha
Lactate
Pyruvate
TCA cycle
B
Nor P17
OIR P17
Relative mRNA levels
Ldha
Pdk1
Pfkfb3
C
Nor P17
OIR P17
PFKFB3
60 kDa
LDHA
37 kDa
PDK1
49 kDa
β-actin
42 kDa
Relative protein level
LDHA
PDK1
PFKFB3
D
normoxia
hypoxia
PFKFB3
60 kDa
LDHA
37 kDa
PDK1
49 kDa
β-actin
42 kDa
Relative protein level
LDHA
PDK1
PFKFB3

Figure S5. PFKFB3 expression is elevated in the OIR model and in hypoxic endothelial cells

A,B: Transcriptomic profiling identified robust upregulation of the glycolytic regulators *Pfkfb3*, *Pdk1*, and *Ldha* in OIR retinas compared with those into normoxic controls, independently validated by RT-qPCR (B).

C,D: Western blot demonstrating increased PFKFB3 expression in OIR retinal lysates (C)and hypoxic HRMECs (D).

Data were analyzed using two-tailed unpaired Student's *t*-test. Data represent mean ± SEM. Significance: *$P < 0.05$, **$P < 0.01$, ****$P < 0.0001$.

Figure S6

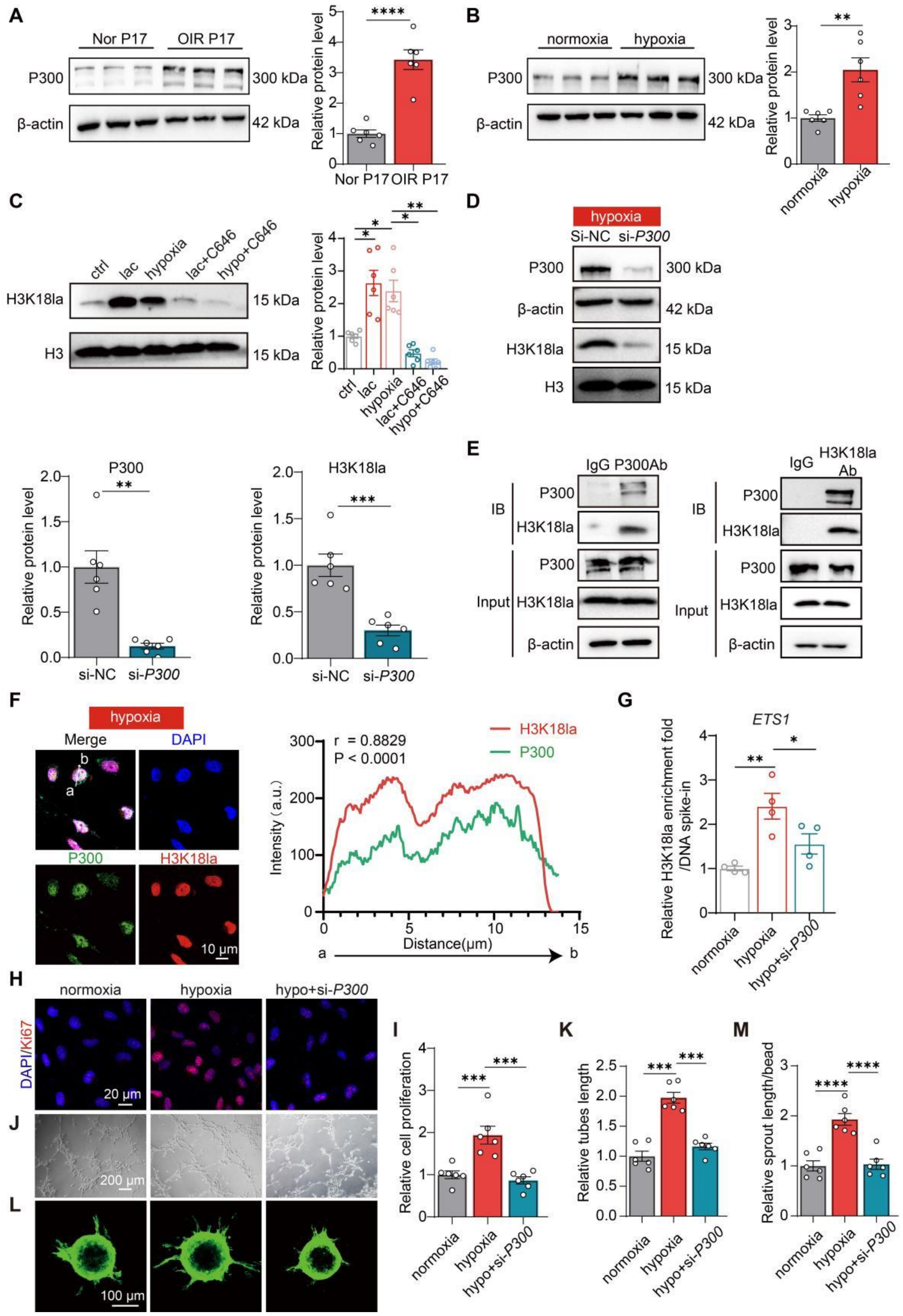
A
Nor P17
OIR P17
P300
300 kDa
β-actin
42 kDa
Relative protein level
****
Nor P17
OIR P17
B
normoxia
hypoxia
P300
300 kDa
β-actin
42 kDa
Relative protein level
**
normoxia
hypoxia
C
ctrl
lac
hypoxia
lac+C646
hypo+C646
H3K18la
15 kDa
H3
15 kDa
Relative protein level
D
hypoxia
Si-NC
si-P300
P300
300 kDa
β-actin
42 kDa
H3K18la
15 kDa
H3
15 kDa
P300
Relative protein level
si-NC
si-P300
H3K18la
***
E
IgG
P300Ab
IB
P300
H3K18la
Input
β-actin
IgG
H3K18la Ab
F
hypoxia
Merge
DAPI
P300
H3K18la
10 μm
r = 0.8829
P < 0.0001
H3K18la
P300
Intensity (a.u.)
Distance(μm)
a
b
G
ETS1
Relative H3K18la enrichment fold /DNA spike-in
normoxia
hypoxia
hypo+si-P300
H
normoxia
hypoxia
hypo+si-P300
DAPI/Ki67
20 μm
J
200 μm
L
100 μm
I
Relative cell proliferation
K
Relative tubes length
M
Relative sprout length/bead
****

Figure S6. P300-mediated H3K18 lactylation drives pathological retinal neovascularization

A: P300 expression in retinas of normoxic (Nor P17) and OIR mice at P17. β-actin loading control (n = 6).

B: Hypoxia-induced (1% O_2, 24 h) P300 upregulation in HRMECs (n = 6).

C:H3K18 lactylation in HRMECs under: vehicle control/lactate (10 mM, 24 h)/hypoxia (1% O_2, 24 h)/ lac + C646 (10 μM)/ hypo + C646 (10 μM) (n = 6).

D: *P300* knockdown efficiency and H3K18la suppression using siRNA (50 nM) in hypoxic HRMECs (n = 6).

E: Co-immunoprecipitation validating P300-H3K18la interaction (n = 3).

F: Confocal imaging of P300-H3K18la colocalization:P300 (green)/H3K18la (red). Pearson's r = 0.8829. Scale bar: 10 μm.

G:Genome-wide H3K18la occupancy maps generated by CUT&RUN-qPCR in HRMECs subjected to normoxia (control), hypoxia (1% O_2, 24 h), or hypoxia with *P300* knockdown (n = 4).

H-M: Phenotypic recovery assays after post-*P300* inhibition under hypoxia: H,I: Ki67 proliferation; J,K: Tube formation; L,M: Spheroid sprouting (n = 6; scale bars labeled).

Data represent mean ± SEM. Unpaired two-tailed Student's *t*-tests (A,B, and D) or one-way ANOVA with Tukey's multiple comparison test (C, G, I, K, and M) were performed. Significance: *$P < 0.05$, **$P < 0.01$, ***$P < 0.001$, ****$P < 0.0001$.

Figure S7

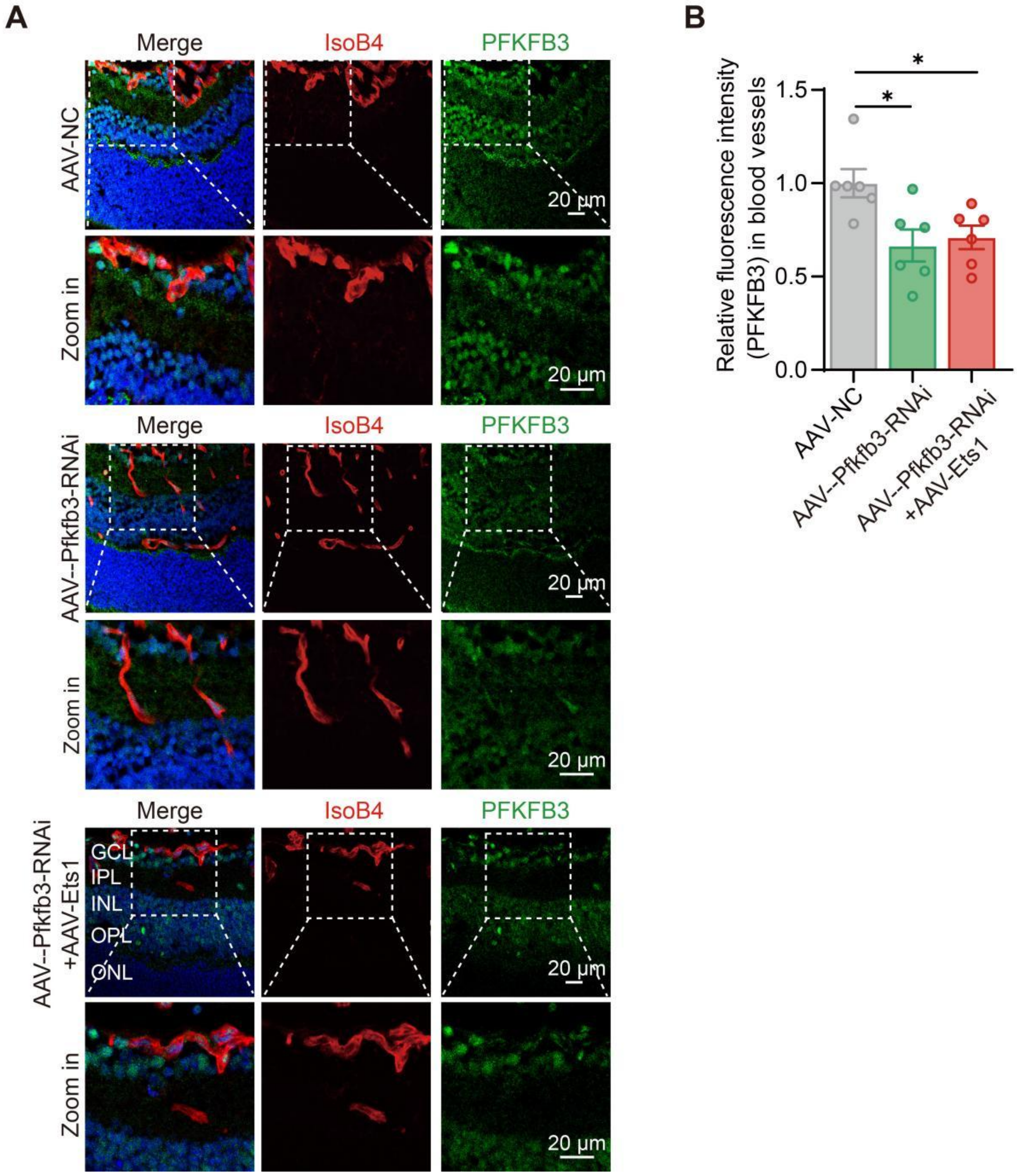
A
Merge
IsoB4
PFKFB3
AAV-NC
Zoom in
AAV--Pfkfb3-RNAi
AAV--Pfkfb3-RNAi
+AAV-Ets1
GCL
IPL
INL
OPL
ONL
20 μm
B
Relative fluorescence intensity
(PFKFB3) in blood vessels
0.0
0.5
1.0
1.5
*
*
AAV-NC
AAV--Pfkfb3-RNAi
AAV--Pfkfb3-RNAi
+AAV-Ets1

Figure S7. PFKFB3 signals are diminished in knockdown retinas

A,B: Quantitative immunofluorescence demonstrating reduced PFKFB3 signal intensity in *Pfkfb3*-knockdown retinas. Scale bar: 20 µm (n = 6).

Supplementary Table 1:The sequences of siRNA

siRNA	Sequence (5'-3')
si*ETS1-a* (Homo sapiens)	CGCUAUACCUCGGAUUACU
si*ETS1-b* (Homo sapiens)	CCACUAUUAACUCCAAGCA
si-*P300* (Homo sapiens)	CCGGUGAACUCUCCUAUAATT
si*PFKFB3* (Homo sapiens)	GAAGAGGATCAGTTGCTATGATT

Supplementary Table 2:Antibodies

Target antigen	Vendor or Source	Catalog#	Working concentration	Persistent ID/URL
PFKFB3	ABclonal	A3934	1/1000(WB)	https://abclonal.com/catalog-antibodies/PFKFB3RabbitmAb/A3934
PDK1/PDHK1	ABclonal	A0834	1/1000(WB)	https://abclonal.com/catalog-antibodies/PDK1PDHK1RabbitpAb/A0834
LDHA	ABclonal	A1146	1/1000(WB)	https://abclonal.com/catalog-antibodies/LDHARabbitpAb/A1146
Anti-L-Lactyl-Histone H3(Lys18) Rabbit mAb	PTM BIO	PTM-1406RM	1/2000(WB) 1/500(IF)	https://ptmbio.com/products/anti-l-lactyl-histone-h3-lys18-rabbit-mab/PTM-1406RM.htm
Anti-L-Lactyl-Histone H3(Lys14) Rabbit mAb	PTM BIO	PTM-1414	1/1000(WB)	https://ptmbio.com/products/anti-l-lactyl-histone-h3-lys14-rabbit-pab/PTM-1414.htm
Anti-L-Lactyl-Histone H4(Lys5) Rabbit mAb	PTM BIO	PTM-1407RM	1/1000(WB)	https://ptmbio.com/products/anti-l-lactyl-histone-h4-lys5-rabbit-mab/PTM-1407RM.htm
Anti-L-Lactyl-Histone H4(Lys8) Rabbit mAb	PTM BIO	PTM-1415RM	1/1000(WB)	https://ptmbio.com/products/anti-l-lactyl-histone-h4-lys8-rabbit-mab/PTM-1415RM.htm

Anti-L-Lactyl-Histone H4(Lys12) Rabbit mAb	PTM BIO	PTM-1411RM	1/1000(WB)	https://ptmbio.com/products/anti-l-lactyl-histone-h4-lys12-rabbit-mab/PTM-1411RM.htm
Anti-L-Lactyl-Histone H3(Lys18) Rabbit mAb	PTM BIO	PTM-1427RM	1/50 (CUT&Tag)	https://www.ptmbio.com/search/list.htm?searchtype=product&keyword=PTM-1427RM
Anti-Histone H3/HIST3H3 Monoclonal Antibody	Solarbio	K200033M	1/1000(WB)	https://www.solarbio.com/goodsInfo?id=9279
Anti-L-Lactyl Lysine Rabbit mAb	PTM BIO	PTM-1401RM	1/1000(WB) 1/500(IF)	https://ptmbio.com/products/anti-l-lactyllysine-rabbit-mab/PTM-1401RM.htm
Ets-1(C-4)	SANTA CRUZ BIOTECHNOLOGY	sc-55581	1/1000(WB) 1/200(IF)	https://www.scbt.com/zh/p/ets-1-antibody-c-4
Ki67	Abcam	ab16667	1/200(IF)	https://www.abcam.cn/products/primary-antibodies/ki67-antibody-sp6-ab16667.html
DAPI	Abcam	ab104139	IF	https://www.abcam.cn/products/media/mounting-medium-with-dapi-aqueous-fluoroshield-ab104139.html
β-actin	Proteintech	66009-1-Ig	1/1000(WB)	https://www.ptgcn.com/products/Pan-Actin-Antibody-

				66009-1-Ig.htm
Anti-Histone H3 (acetyl K27) - ChIP Grade	Abcam	ab4729	1/50 (CUT&Tag)	https://www.abcam.cn/products/primary-antibodies/histone-h3-acetyl-k27-antibody-chip-grade-ab4729.html
PFKFB3	Abcam	ab181861	1/200(IF)	https://www.abcam.cn/products/primary-antibodies/pfkfb3-antibody-epr12594-ab181861.html

Supplementary Table3:Primer sequences used in RT-qPCR.

	**Gene**	**Primer**	**Primer Sequence (5′-3′)**	**Length**
Homo sapiens	*ETS1*	F	GCCAGTCATCTTTCAACAG	126
		R	CACGGTCCCGCACATAG	
	ACTB	F	AGCACAGAGCCTCGCCTTT	70
		R	ATCATCATCCATGGTGAGCTGG	
	HK2	F	AGGTCCTGATGCGGTTGG	154
		R	CGCTCCTCGCCTTTGTTC	
	CXCR4	F	CCACCAACAGTCAGAGGC	101
		R	AAAGATGAAGTCGGGAATAG	
	TGM2	F	ACAAATCCATCAACCGTTCC	160
		R	GCCAGTTTGTTCAGGTGGTT	

	**Gene**	**Primer**	**Primer Sequence (5′-3′)**	**Length**
Mus musculus	*Ets1*	F	GAGATCCTGCAGAAAGAGGATG	178
		R	GGAGCGTCTGATAGGACTCTGT	
	Actb	F	GGCTGTATTCCCCTCCATCG	154
		R	CCAGTTGGTAACAATGCCATGT	
	Pfkfb3	F	GATCTGGGTGCCCGTCGATCACCG	147
		R	CAGTTGAGGTAGCGAGTCAGCTTC	
	Ldha	F	TGTCTCCAGCAAAGACTACTGT	155
		R	GACTGTACTTGACAATGTTGGGA	
	Pdk1	F	AGAGTCTGTCGATCTTCCTCA	96
		R	CGATTCAAGTTCACGTCACAC	

	Tgm2	F	GCTGGACCAACAGGACAATGT	85
		R	CTCTAGGCTGAGACGGTACAG	
	Hk2	F	TGATCGCCTGCTTATTCACGG	112
		R	AACCGCCTAGAAATCTCCAGA	
	Cxcr4	F	GAAGTGGGGTCTGGAGACTAT	125
		R	TTGCCGACTATGCCAGTCAAG	

Supplementary Table 4:Primer sequences used in CUT&RUN-qPCR assays

Sites	Primer Sequence (5´-3´)
ETS1(Homo sapiens)	Forward primer：GCCCGTGGATGGTCTAAT
	Reverse primer：GATGTGGGTGTCGCTGGT
